# Cell fate specification in the lingual epithelium is controlled by antagonistic activities of Sonic hedgehog and retinoic acid

**DOI:** 10.1371/journal.pgen.1006914

**Published:** 2017-07-17

**Authors:** Maha El Shahawy, Claes-Göran Reibring, Cynthia L. Neben, Kristina Hallberg, Pauline Marangoni, Brian D. Harfe, Ophir D. Klein, Anders Linde, Amel Gritli-Linde

**Affiliations:** 1 Department of Oral Biochemistry, Institute of Odontology, Sahlgrenska Academy at the University of Gothenburg, Göteborg, Sweden; 2 Program in Craniofacial Biology and Department of Orofacial Sciences, University of California San Francisco, San Francisco, CA, United States of America; 3 Department of Molecular Genetics and Microbiology, University of Florida College of Medicine, Gainesville, FL, United States of America; 4 Department of Pediatrics and Institute for Human Genetics, University of California San Francisco, San Francisco, CA, United States of America; University of Helsinki, FINLAND

## Abstract

The interaction between signaling pathways is a central question in the study of organogenesis. Using the developing murine tongue as a model, we uncovered unknown relationships between Sonic hedgehog (SHH) and retinoic acid (RA) signaling. Genetic loss of SHH signaling leads to enhanced RA activity subsequent to loss of SHH-dependent expression of *Cyp26a1* and *Cyp26c1*. This causes a cell identity switch, prompting the epithelium of the tongue to form heterotopic minor salivary glands and to overproduce oversized taste buds. At developmental stages during which *Wnt10b* expression normally ceases and *Shh* becomes confined to taste bud cells, loss of SHH inputs causes the lingual epithelium to undergo an ectopic and anachronic expression of *Shh* and *Wnt10b* in the basal layer, specifying *de novo* taste placode induction. Surprisingly, in the absence of SHH signaling, lingual epithelial cells adopted a Merkel cell fate, but this was not caused by enhanced RA signaling. We show that RA promotes, whereas SHH, acting strictly within the lingual epithelium, inhibits taste placode and lingual gland formation by thwarting RA activity. These findings reveal key functions for SHH and RA in cell fate specification in the lingual epithelium and aid in deciphering the molecular mechanisms that assign cell identity.

## Author summary

Knowledge of the biological mechanisms controlling cell fate specification is of paramount importance for cell-based therapies. Sonic hedgehog (SHH) and retinoic acid (RA) pathways play key roles in development and disease. The role of SHH during *in vivo* tongue development is a subject of great interest, and whether RA signaling has any function in the developing tongue is unknown. The tongue is covered by a mucosa made of lingual epithelium and lingual mesenchyme. Various structures, including mechanosensory filiform papillae, gustatory papillae harboring taste buds, and minor salivary glands, arise from the epithelium, but how these entities are specified remains unclear. Here we show that in the mesenchyme SHH signaling drives growth and morphogenesis, whereas in the epithelium, SHH controls patterning and cell fate specification. We demonstrate that SHH inhibits taste placode and lingual gland formation by antagonizing RA inputs. We also show that loss of SHH signaling elicits Merkel cell formation in the lingual epithelium, a tissue normally bereft of Merkel cells. This is at odds with the hairy epidermis where Merkel cell specification has been shown to be SHH-dependent. Our study establishes SHH and RA as key players in the control of cell identity within the lingual epithelium.

## Introduction

**Fig 1:**
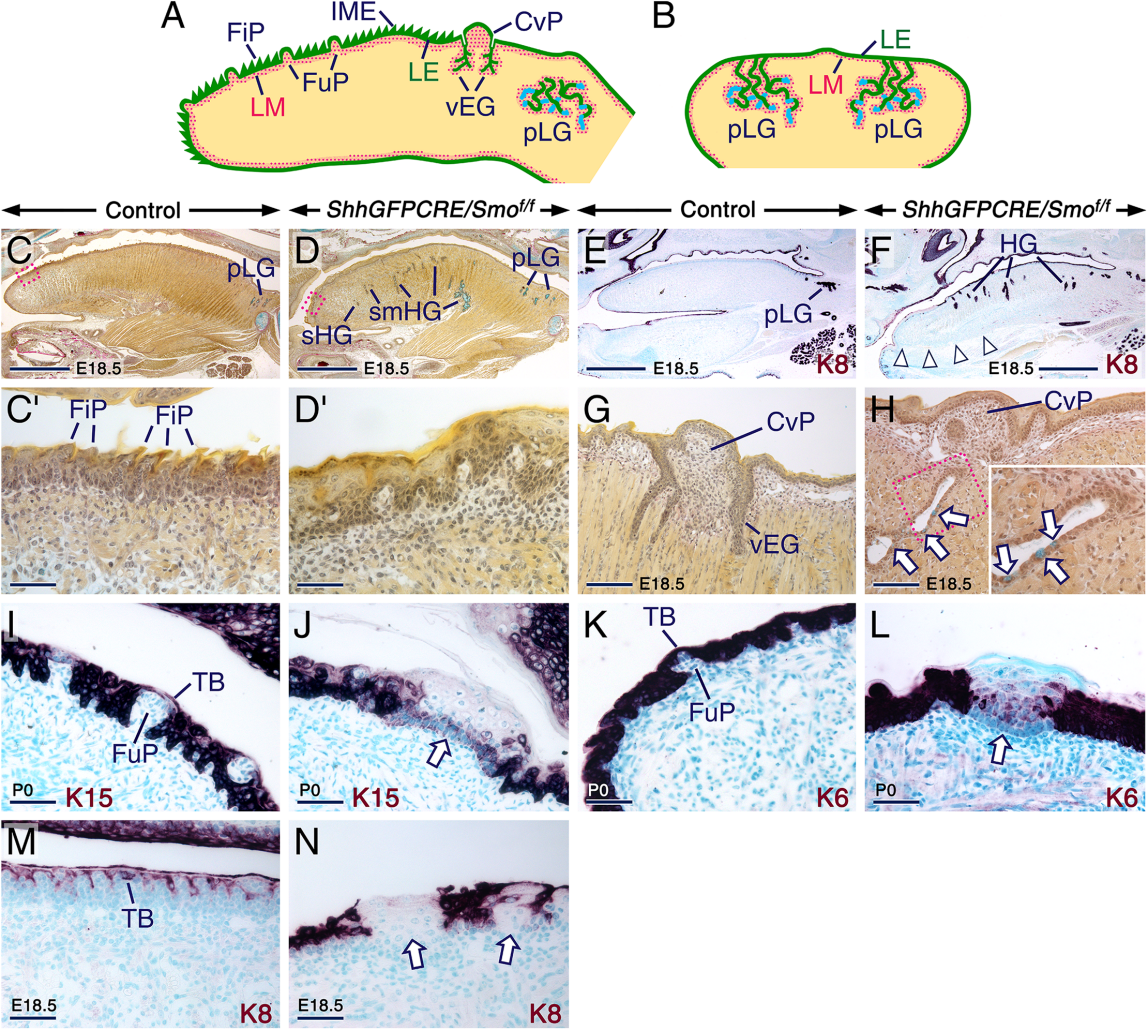
Loss of *Smo* in the lingual epithelium engenders sero-mucous glandular metaplasia amidst focal differentiation defects. (**A,B**) Schematics depicting midsagittal (A) and frontal (B; across the pharyngeal tongue) sections of the murine tongue at E18.5 to postnatal day P0. (**C-N**) Tongue sections from control and *ShhGFPCRE/Smo*^*f/f*^ mutants. (**C-D’**) Parasagittal sections of E18.5 control (C) and mutant (D) tongues after alcian blue van Gieson staining. (C’) and (D’) are enlarged images of the boxed areas in (C) and (D), respectively. The oral tongue of mutants exhibits epithelial hyperplasia, lacks filiform papillae (FiP), and displays serous (sHG) and sero-mucous (smHG; mucous cells stain blue) heterotopic glands. (**E,F**) Anti-Keratin 8-stained (K8; purple) parasagittal sections of E18.5 control (E) and mutant (F) tongues. The oral tongue of the mutant shows K8-positive heterotopic glands (HG). Adhesion between the tongue and floor of the oral cavity in the mutant (arrowheads in F). (**G,H**) Alcian blue van Gieson-stained sections across the circumvallate papilla (CvP) of E18.5 control (G) and mutant (H) tongues. The mutant shows overgrown von Ebner’s glands (vEG) exhibiting mucous metaplasia (arrows in H). The inset in (H) is an enlarged image of the boxed area in (H). (**I-N**) Frontal (I,J) and parasagittal (K-N) tongue sections from controls (I,K,M) and mutants (J,L,N) at P0 (I-L) and E18.5 (M,N) immunostained for Keratin 15 (K15), Keratin 6 (K6) and K8 (purple). The mutant tongues exhibit epithelial foci with weak or no K15, K6, and K8 staining (arrows in J,L and N). CvP, circumvallate papilla; FiP, filiform papillae; FuP, fungiform papillae; IME, intermolar eminence; LE, lingual epithelium; LM, lingual mesenchyme; TB, taste bud; pLG, posterior lingual glands. Scale bars: 1 mm (C-F), 100 μm (G,H), and 50 μm (C’,D’, I-N).

The tongue is a muscular organ that plays critical roles in mastication, speech and taste, and the regulatory mechanisms that specify the diverse cell types and structures of the tongue are of great interest. The mature tongue (Fig 1A and 1B) is covered by a mucosa made of lingual epithelium (LE) and lingual mesenchyme (LM). The dorsal LE of the anterior 2/3 of the tongue (oral tongue) is a stratified, squamous epithelium primarily comprised of mechanosensory filiform papillae. The gustatory units of the LE, the taste buds (TBs), develop in three different types of papillae: fungiform papillae, foliate papillae, and circumvallate papillae. Fungiform papillae house a single TB and are distributed between filiform papillae over the dorsal surface of the oral tongue, whereas foliate papillae house several TBs and develop posteriorly at the lateral edges of the oral tongue. In rodents, a single circumvallate papilla harbouring numerous TBs forms at the junction between the oral tongue and the posterior 1/3 of the tongue, known as pharyngeal tongue [1]. The LE also produces minor salivary glands of a mixed sero-mucous type in the pharyngeal tongue, as well as a purely serous type, von Ebner’s glands, which arise from the epithelium of the circumvallate papilla [2,3].

The distribution of mature taste papillae depends on coordinated signaling events during early stages of tongue development [4]. From embryonic day (E) 10.5-E11.5, *Shh* is expressed throughout the LE and signals both within the LE and to the LM [5–8]. At E12.5, fungiform papilla development is heralded by fungiform placodes (FPs), localized thickenings of the LE made of post-mitotic TB precursors expressing *Shh*, *Wnt10b*, and *Bmps*. At E12.5 to E14, the interplacodal epithelium is devoid of *Shh* expression, which is restricted to taste placodes. Canonical signaling through these pathways is critical for regulating the size, number, and spacing of fungiform placodes [4]. As the tongue grows, new FPs develop until E14.5, at which time fungiform papilla morphogenesis begins and differentiating TBs become morphologically and molecularly visible at the tip of fungiform papillae. After E14.5 and during early postnatal life, only developing TB cells within gustatory papillae produce SHH [4].

Several components of the Hedgehog signaling pathway play key roles to ensure properly calibrated spatio-temporal Hedgehog inputs [8–11]. Upon ligand binding to the Hedgehog receptor Patched (PTCH1), Smoothened (SMO), an obligatory Hedgehog transducer, translocates to the primary cilium, unleashing a signaling cascade culminating in transcriptional regulation of Hedgehog target genes by GLI proteins [10]. *Gli1* and *Ptch1* are themselves direct targets of Hedgehog signaling, and hence their expression enables identification of cells responding to Hedgehog signals. Loss of SMO function abrogates all Hedgehog signaling even in the presence of copious amounts of ligands [9].

Studies of tongue organ cultures have shown that pharmacological inhibition or activation of SHH signaling causes increased or decreased size and number of FPs, respectively, indicating that SHH inhibits FP formation [12–15]. However, whether and how SHH performs such a function *in vivo* is unknown [6,16]. Moreover, it remains unclear which lingual tissue SHH acts upon, as *in vitro* manipulations may disrupt the LE and LM which are both SHH-responsive [5,6,8]. Furthermore, how different lingual structures such as TBs and glands are specified within a seemingly homogeneous epithelial sheet remains a critical open question. That SHH signaling is active in both the oral and pharyngeal tongue during the earliest steps of tongue formation [6,8] suggests this molecule plays a yet unknown role in cell fate specification within the LE.

To address these issues, we utilized mouse genetics to modulate SHH signaling in the LE, LM, or both. We found that SHH plays a dual role during tongue development by acting in the LM to control growth and morphogenesis and in the LE to control patterning and cell fate determination. We revealed an unknown, highly sensitive balance between SHH and retinoic acid (RA) signaling that regulates the specification of taste placodes and glands. Upon loss of SHH signaling, the augmented RA inputs direct the LE to (1) form minor salivary glands in the oral tongue, (2) overproduce oversized FPs and TBs, and (3) sustainably generate taste placodes. We found that both FP specification and antero-posterior patterning of the LE are regulated by antagonizing activities of SHH and RA. We show that SHH antagonizes RA signaling by maintaining expression of *Cyp26a1* and *Cyp26c1*. Unexpectedly, we found that in the absence of SHH signaling, the LE aberrantly produced Merkel cells. Finally, we identified two novel TB markers, Homer1 and RALDH1.

## Results and discussion

### Glandular metaplasia, epithelial hyperplasia, and failure of filiform papilla formation upon loss of SMO in the lingual epithelium

To decipher the function of SHH in patterning of and cell type specification in the LE, we first irreversibly ablated SHH signaling in epithelial cells that express *Shh* and their progeny by *ShhGFPCRE*-mediated removal of *Smo* in mutants carrying *Smo* floxed (f) alleles (*ShhGFPCRE/Smo*^*f/f*^). The *ShhGFPCRE/Smo*^*f/f*^ mutants die shortly after birth due to unknown causes. Littermates not carrying the *ShhGFPCRE* knockin and/or the *Smo* floxed alleles and heterozygotes, which are viable and phenotypically normal, were used as controls. Tongues from embryos carrying the *R26R* transgene (*ShhGFPCRE/R26R*) showed robust CRE activity in the LE (S1A–S1D Fig). Probes targeting *Ptch1* and *Gli1*, which are readouts of Hedgehog activity [6,9,17–19], confirmed the absence of *Ptch1* and *Gli1* transcripts in the *ShhGFPCRE/Smo*^*f/f*^ LE, indicating efficient *Smo* ablation in this tissue. By contrast, and as expected, the LM retained endogenous hedgehog signaling (S1E–S1H’ Fig). Consistent with these findings, RT-qPCR revealed downregulation of *Ptch1* in *ShhGFPCRE/Smo*^*f/f*^ tongues (S1I Fig).

To assess the impact of epithelial SMO ablation on tongue development, we compared control and *ShhGFPCRE/Smo*^*f/f*^ tongues at E17.5, E18.5, and shortly after birth (postnatal day P0). Alcian blue van Gieson staining and immunohistochemistry for Keratin 8 (K8) revealed striking defects within the LE and its derivatives in the *ShhGFPCRE/Smo*^*f/f*^ cohort with 100% penetrance. Specifically, the oral tongue, a territory normally bereft of glands, exhibited heterotopic serous and sero-mucous glands (Fig 1C–1F). Furthermore, the LE was hyperplastic, lacked filiform papillae (Fig 1C’–1D’), and displayed overgrown von Ebner’s glands transformed into a sero-mucous type (Fig 1G and 1H; see also S6 Fig), as well as epithelial foci virtually devoid of K8, Keratin 6, and Keratin 15 immunostaining (Fig 1I–1N). 7.6% of mutants also showed adhesions between the LE and epithelia of the floor of the oral cavity (Fig 1F). Taken together, these findings indicate that an altered antero-posterior patterning and differentiation program in the SMO-deficient LE is translated into development of glands in the oral tongue, abnormal development of von Ebner’s glands, and formation of epithelial regions exhibiting a molecular signature different from that of the normal LE.

### The *ShhGFPCRE/Smo*^*f/f*^ tongue undergoes Merkel cell metaplasia

**Fig 2:**
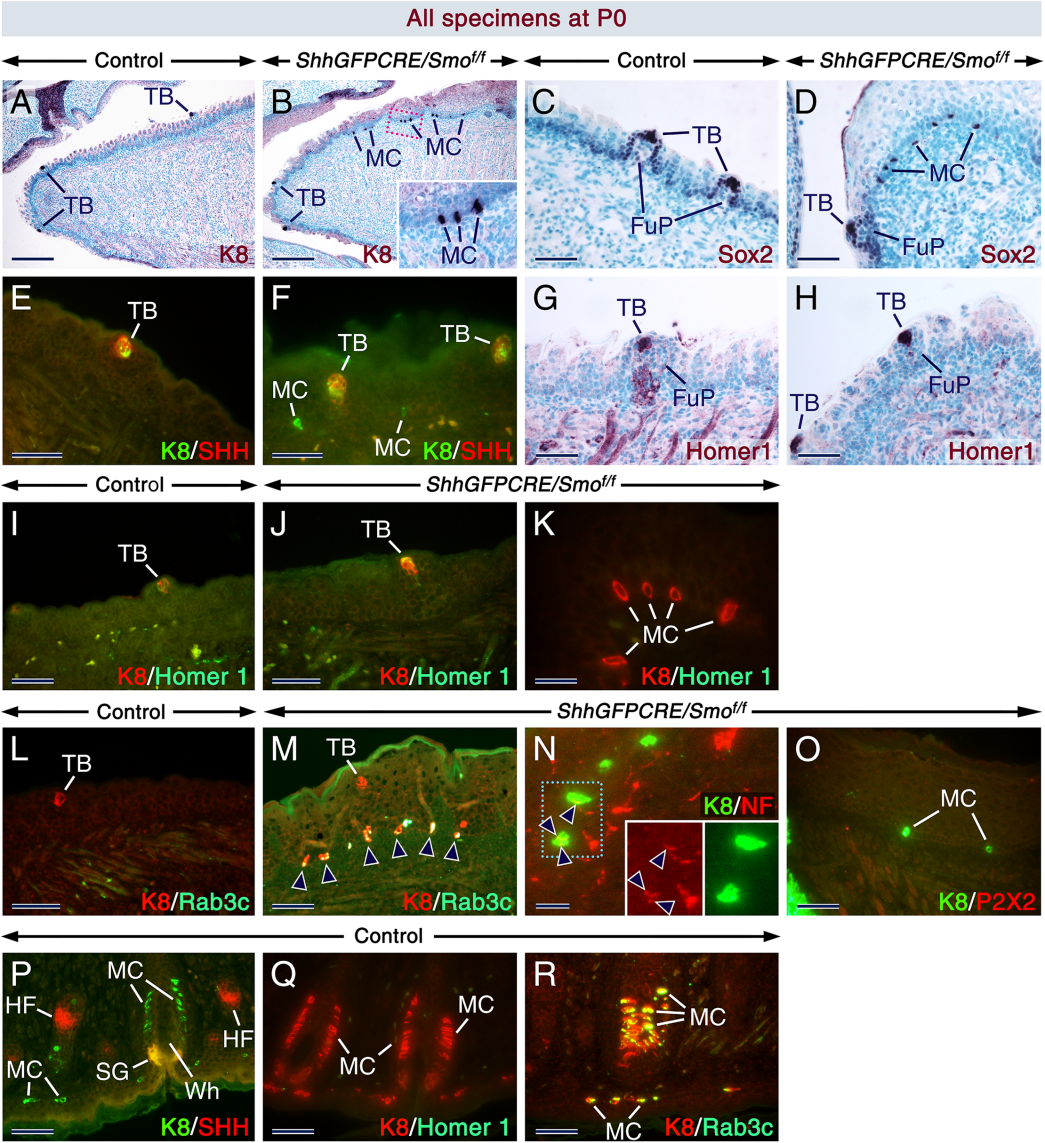
The *ShhGFPCRE/Smo*^*f/f*^ lingual epithelium undergoes Merkel cell metaplasia. (**A-O**) Parasagittal tongue sections from newborn (P0) controls and *ShhGFPCRE/Smo*^*f/f*^ mutants. (**A-D**) Sections of control (A,C) and mutant (B,D) tongues immunostained (purple) for Keratin 8 (K8; A,B) and SOX2 (C,D). The inset in (B) is an enlarged image of the boxed area in (B). The mutant tongues exhibit K8-positive (+) and SOX2+ ectopic Merkel cells (MC) in the epithelial basal layer. Taste buds (TB) are also K8+ and SOX2+. (**E,F**) Sections of control (E) and mutant (F) tongues after double staining for K8 (green) and Sonic Hedgehog (SHH; red). TBs are K8+/SHH+. The K8+ ectopic Merkel cells in the mutant are SHH-negative (–). (**G,H**) Anti-Homer1-stained (purple) sections of control (G) and mutant (H) tongues showing Homer1(+) TBs. (**I-K**) Sections of control (I) and mutant (J,K) tongues after double staining for K8 (red) and Homer1 (green), showing K8+/Homer1+ TBs (I,J) and K8+/Homer1(–) ectopic Merkel cells (K). (**L,M**) Sections of control (L) and mutant (M) tongues after double staining for K8 (red) and Rab3c (green). The ectopic Merkel cells (arrowheads in M) are K8+/Rab3c+, whereas TBs are K8+/Rab3c(–). (**N**) Section across a mutant tongue after double staining for K8 (green) and NF-200 (NF; red) showing ectopic Merkel cells wrapped by NF-200+ neurites (arrowheads). The insets in (N) are non-merged, enlarged images of the boxed area in (N). (**O**) Section of a mutant tongue after double staining for K8 (green) and P2X2 (red) showing that the ectopic Merkel cells are not associated with P2X2+ gustatory neurites. (**P-R**) Sections across the whisker pad of control newborns after double staining for K8/SHH (P), K8/Homer1(Q) and K8/Rab3c (R). Merkel cells of the whisker follicles and adjacent epidermis are K8+/SHH(–), K8+/Homer1(–), and K8+/Rab3c+. FuP, fungiform papillae; HF, hair follicle; SG, sebaceous glands; Wh, whisker follicle. Scale bars: 200 μm (A,B), 50 μm (C-J, L,M, O-R), and 20 μm (K,N).

Closer examination revealed abnormal formation of K8-positive (+) (Fig 2A and 2B) and SOX2+ (Fig 2C and 2D) solitary cells in the basal layer of the LE in all *ShhGFPCRE/Smo*^*f/f*^ mutants examined at E17.5 to P0. We postulated that these cells could be stray TB cells or ectopic epithelial tactile cells called Merkel cells, as both cell types express high levels of *Sox2* and *Krt8* [20–22]. Merkel cells do not express *Shh* (Fig 2P) [21], whereas all TB cells express SHH at least up to the perinatal period (Fig 2E and 2F) [4]. K8/SHH immunostaining revealed absence of SHH+ solitary cells in the basal layer of the *ShhGFPCRE/Smo*^*f/f*^ LE and showed that the K8+ solitary cells were SHH-negative (–) (Fig 2F; S1 Table), suggesting that these were not TB cells. To further assess this notion, we immunostained sections for Homer1, a scaffolding protein found in skeletal muscle and brain [23] which we serendipitously identified as a reliable TB marker in the course of this study; Homer1 expression in the LE of control and *ShhGFPCRE/Smo*^*f/f*^ tongues is not only restricted to the TBs, but also readily detectable in all TB cell types (Fig 2G–2J; S1 Table), differing from other marker proteins that are only expressed in subsets of TB cells [1,4]. Neither orthotopic K8+ Merkel cells in whisker pads (Fig 2Q) [21] nor the K8+ solitary cells in the basal layer of the *ShhGFPCRE/Smo*^*f/f*^ LE (Fig 2K; S1 Table) were Homer1+. Thus, two TB markers, SHH and Homer1, excluded a TB cell type and supported a Merkel cell identity of the solitary cells in the mutant LE. To confirm that these cells were Merkel cells, we examined expression of Rab3c, a protein known to be enriched in Merkel cell cytoplasmic vesicles (Fig 2R) [21]. We found that while the basal layer of the LE in controls as well as K8+ TBs in controls and *ShhGFPCRE/Smo*^*f/f*^ mutants were Rab3c(–), the ectopic K8+ solitary cells within the *ShhGFPCRE/Smo*^*f/f*^ LE were Rab3c+ (Fig 2L and 2M; S1 Table). These data show that the K8+, SOX2+, and Rab3c+ cells were indeed ectopic Merkel cells, indicating that the LE, a tissue normally devoid of Merkel cells in humans and mice, underwent Merkel cell metaplasia upon loss of SMO.

In mice, Merkel cells develop in whisker pads and touch domes of hairy and glabrous skin [24] and become innervated postnatally [24,25]. Remarkably, we found that the majority of the K8+ ectopic Merkel cells in the *ShhGFPCRE/Smo*^*f/f*^ LE were innervated by NF-200+ neurites (Fig 2N; S1 Table), suggesting that they might be functional in this foreign environment. TBs in fungiform papillae are innervated by P2X2+ gustatory axons [26], however, none of the K8+ basally located solitary cells in the *ShhGFPCRE/Smo*^*f/f*^ LE were innervated by P2X2+ axons (Fig 2O; S1 Table), further supporting our conclusion that these cells are ectopic Merkel cells and not TB cells.

Unlike in the glabrous skin where Merkel cells develop in the absence of SHH inputs, Merkel cell specification in the hairy skin is dependent on SHH signaling [27]. Here we showed that in the absence of SMO, a subset of cells in the LE are diverted to a Merkel cell fate. Taken together, these observations suggest a context-dependent requirement for SHH signaling for Merkel cell specification. Polycomb genes have been shown to repress *Sox2* which encodes a crucial factor for Merkel cell specification in the epidermis [28]. However, the LE expresses *Sox2* mRNA and protein from early stages of tongue development onwards [22,29], and the ectopic Merkel cells in the *ShhGFPCRE/Smo*^*f/f*^ LE express SOX2 protein. These observations likely indicate that Polycomb genes are not effectors of the repression of Merkel cell formation in the LE. TBs and Merkel cells both degenerate after denervation [30,31] and share common markers such as *Krt8*, K20, *Sox2*, *Advillin*, *Satb2*, *Cpe*, Claudins 6/7, *Mash1*, *Hes6*, and *Snap25* [1,21,22,32–34]. This raises the possibility that a subset of TB progenitors converted into Merkel cells in the SMO-deficient LE.

### Overproduction of oversized fungiform placodes upon epithelial loss of SMO

**Fig 3:**
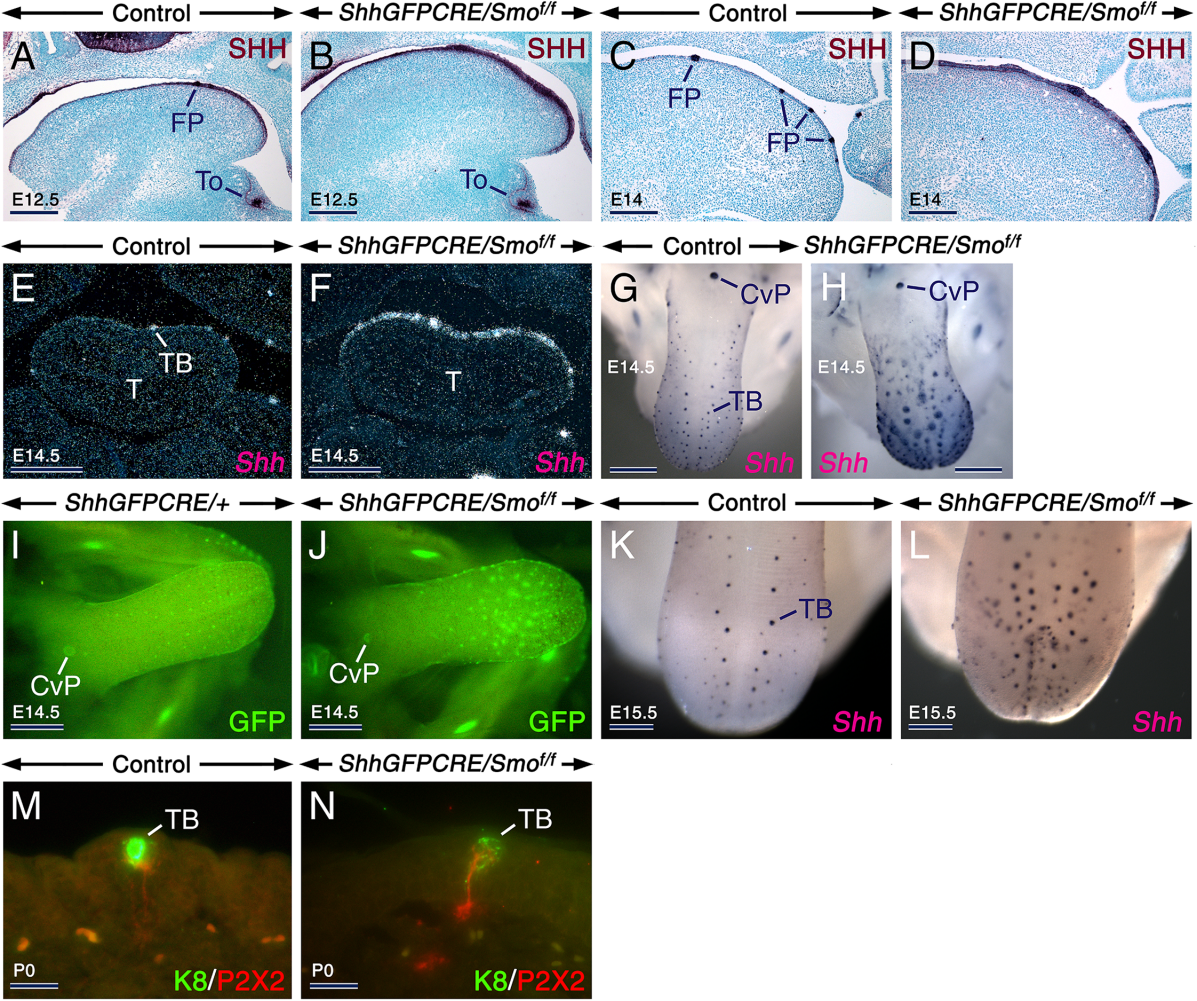
The *ShhGFPCRE/Smo*^*f/f*^ mutant tongues show expansion of *Shh* expression in the lingual epithelium, and develop innervated taste buds. (**A-D**) Anti-Sonic hedgehog-stained (SHH; purple) parasagittal tongue sections from controls (A,C) and mutants (B,D) at E12.5 (A,B) and E14 (C,D) showing abnormally expanded SHH staining in the lingual epithelium (LE) of the E14 mutant. (**E,F**) Dark-field images of frontal tongue sections from E14.5 control (E) and mutant (F) embryos after *Shh in situ* hybridization showing abnormally expanded *Shh* expression in the LE of the mutant. (**G-L**) E14.5 (G-J) and E15.5 (K,L) control (G,I,K) and mutant (H,J,L) tongues after *in situ* hybridization (dark blue/brown; G,H,K,L) and green fluorescent protein (GFP) imaging (I,J) showing *Shh* expression. The mutant tongues exhibited abnormally enlarged *Shh*+ spots, some of which developed ectopically along the tongue midline (H,J,L). (**M,N**) Parasagittal tongue sections from control (M) and mutant (N) newborns (P0) after double staining for Keratin 8 (K8; green) and P2X2 (red) showing innervated TBs. CvP, circumvallate papilla; FP, fungiform placode; T, tongue; TB, taste bud; To, tooth bud. Scale bars: 500 μm (E-J), 300 μm (K,L), 200 μm (A-D), and 25 μm (M,N).

*In vitro* modulation of SHH signaling in the developing rodent tongue impinges upon FP patterning [12–15], however, it is currently unclear whether this is due to altered SHH activities in the LE, LM, or both. Furthermore, whether SHH has a role in FP patterning *in vivo* is still unknown. To address these points, we analyzed the effects of loss of epithelial SMO on FP development by assessing the expression patterns of *Shh* mRNA and protein, an established taste placode marker. At E12.5, the LE and LM showed robust and weak SHH immunostaining, respectively, in both control and *ShhGFPCRE/Smo*^*f/f*^ tongues. The staining of the LM and interplacodal epithelium was the result of protein diffusion from taste placodes which produce SHH (Fig 3A and 3B). At E14, instead of being restricted to FPs as in control tongues, SHH staining was expanded in the LE of the *ShhGFPCRE/Smo*^*f/f*^ tongues (Fig 3C and 3D). At E14.5 FP induction normally ceases, and *Shh* transcripts become confined to developing TBs [4]. While at E14.5 and E15.5 the control tongues showed this expected *Shh* expression pattern, the *ShhGFPCRE/Smo*^*f/f*^ tongues displayed a remarkable expansion of *Shh* expression (Fig 3E–3H) and overproduced enlarged *Shh*-expressing spots (FPs and/or TBs), some of which developed ectopically (Fig 3G–3L). Quantification in E15.5 mutant and control tongues revealed increased numbers of *Shh*+ spots in the mutants (147 ± 8 in mutants *vs* 101 ± 7 in controls; mean ± SD; *P* = 0.000). Thus, loss of SMO in the LE not only caused Merkel cell and glandular metaplasiae, but also engendered mispatterning of FPs as a result of their overproduction. Despite displaying FP patterning defects, the *ShhGFPCRE/Smo*^*f/f*^ tongues developed K8+ TBs that were innervated by P2X2 neurites (Fig 3M and 3N; S1 Table).

### Temporal requirement for SHH inputs in the developing tongue for growth, morphogenesis, patterning and cell type specification

**Fig 4:**
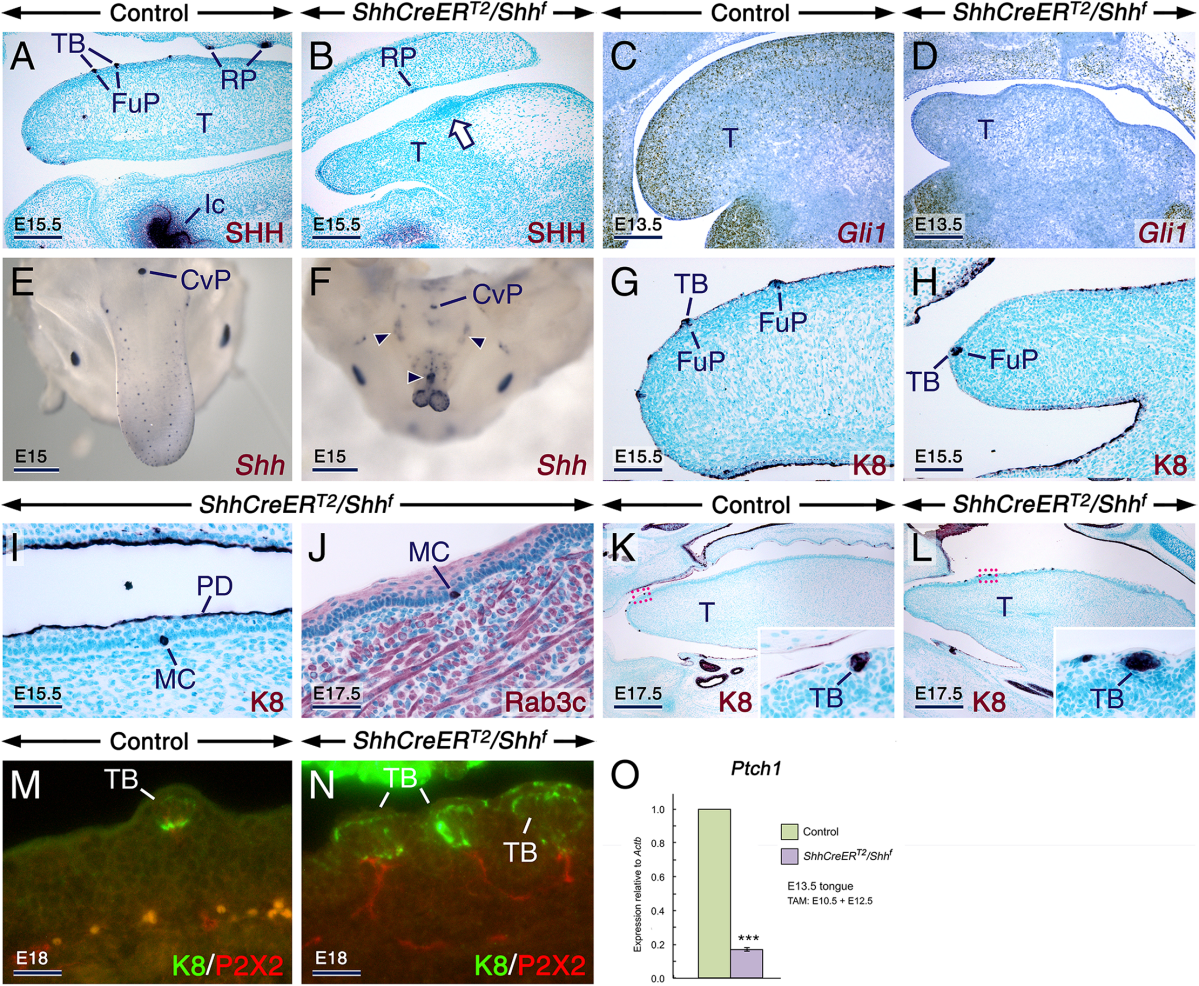
Early loss of SHH signaling in the tongue impinges upon growth and morphogenesis but is conducive to taste bud differentiation. (**A-N**) Tongues and parasagittal tongue sections from control and *ShhCreER*^*T2*^*/Shh*^*f*^ mutant embryos first exposed to tamoxifen at E10.5. (**A**,**B**) Anti-Sonic hedgehog-stained (SHH; dark purple) sections of E15.5 control (A) and mutant (B) tongues showing focal epithelial hyperplasia (arrow in B) and severely decreased SHH immunostaining in the mutant. (**C**,**D**) *Gli1 in situ* hybridization (brown) in sections from E13.5 control (C) and mutant (D) tongues showing severe downregulation of *Gli1* expression in the mutant. (**E,F**) E15 control (E) and mutant (F) tongues after *in situ* hybridization with a riboprobe targeting both deleted (exon2) and non-deleted (exon1) *Shh*-coding sequences (dark purple). Abnormally small mutant tongue with a bifid tip, and exhibiting oversized *Shh*+ spots (arrowheads in F). (**G-L**) Sections of E15.5 (G-I) and E17.5 (J-L) control (G,K) and mutant (H-J,L) tongues immunostained (dark purple) for keratin 8 (K8; G-I, K,L) and Rab3c (J). The insets in (K) and (L) are enlarged images of the boxed areas in (K) and (L), respectively. The control and mutant tongues show K8+ taste buds (TB) in fungiform papillae (FuP), and the mutant tongues exhibit K8+ and Rab3c+ ectopic Merkel cells (MC). (**M,N**) Tongue sections from E18 control (M) and mutant (N) embryos after K8 (green) and P2X2 (red) double staining showing innervated TBs. (**O**) RT-qPCR analysis for *Ptch1* relative to *Actb* (β-actin) in tongues from E13.5 controls (n = 6) and mutants (n = 6) first exposed to tamoxifen (TAM) at E10.5. Severely decreased *Ptch1* levels in the mutant tongues as compared to the controls (*P* = 0.0000; mean values ± SD). PD, periderm; CvP, circumvallate papilla; Ic, incisor; RP, rugae palatinae; T, tongue. Scale bars: 500 μm (E,F,K,L), 200 μm (A-D), 100 μm (G,H), 50 μm (I,J), and 25 μm (M,N).

Having uncovered a stringent requirement for epithelial SHH signaling for patterning and differentiation of the LE, we sought to decipher the role of SHH inputs in the LE and LM by assessing the impact of loss of SHH production. We generated *ShhCreER*^*T2*^*/Shh*^*f*^ mutant mice carrying both a *Shh* floxed allele and the tamoxifen (TAM) -inducible *ShhCreER*^*T2*^ knockin allele. Without exposure to TAM, the *ShhCreER*^*T2*^*/Shh*^*f*^ mice are viable and phenotypically normal. Ablation of *Shh* in the mutants was induced at E10.5. Immunostaining and *in situ* hybridization confirmed that TAM exposure led to a dramatic decrease of SHH protein in the LE and severe downregulation of *Gli1* expression in the LE and LM (Fig 4A–4D) of the *ShhCreER*^*T2*^*/Shh*^*f*^ tongues, indicating loss of SHH signaling. This was further confirmed by RT-qPCR for *Ptch1* (Fig 4O). Upon dissection, we found that relative to their control littermates, the E10.5 TAM-induced *ShhCreER*^*T2*^*/Shh*^*f*^ mutants developed abnormally small tongues (microglossia) with a bifid tip as well as epithelial foci of squamous hyperplasia (Fig 4A–4F). Of note, bifid tip of the tongue has been described in patients with Ellis-van Creveld syndrome, which is caused by mutations of the *EVC* and *EVC2* genes that lead to decreased Hedgehog signaling [35–38].

*In situ* hybridization with a *Shh* riboprobe targeting both deleted (exon2) and non-deleted (exon1) *Shh*-coding sequences [39] revealed enlarged *Shh*+ spots, likely TBs or FPs, some of which were ectopically located along the midline of the E10.5 TAM-induced *ShhCreER*^*T2*^*/Shh*^*f*^ tongues (Fig 4E and 4F). Furthermore, these tongues formed K8+ TBs (Fig 4H and 4L) innervated by P2X2+ neurites (Fig 4M and 4N; S2 Table). However, unlike the *ShhGFPCRE/Smo*^*f/f*^ tongues, the E10.5 TAM-induced *ShhCreER*^*T2*^*/Shh*^*f*^ tongues were exempt from glandular metaplasia, *i*.*e*. no heterotopic glands developed in the oral tongue (Fig 4L), and displayed only a few scattered ectopic Merkel cells (Fig 4I and 4J). Thus, SHH is required for growth and morphogenesis of both the LE and LM, but it is dispensable for FP induction.

**Fig 5:**
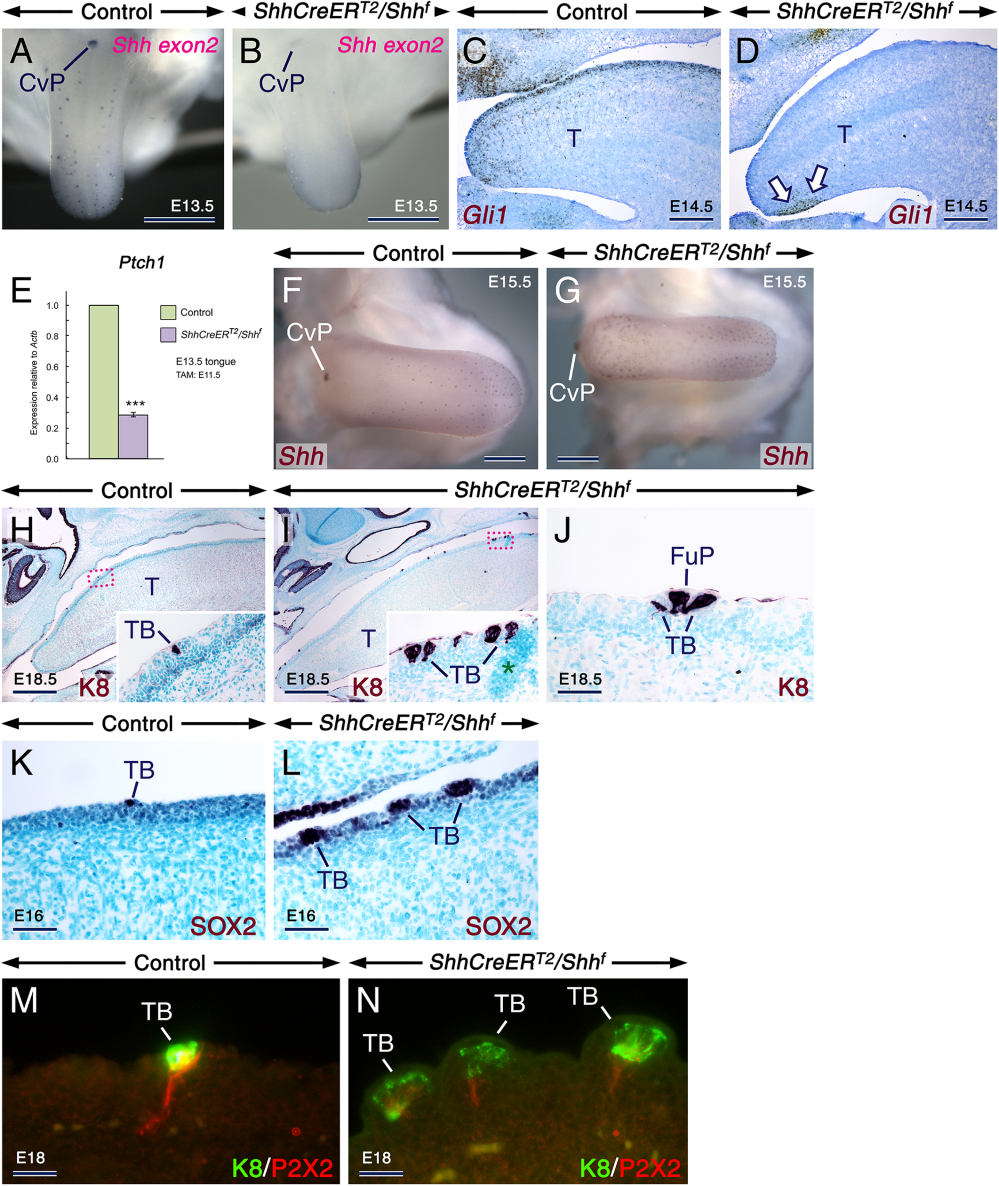
Tamoxifen induction at E11.5 causes overproduction of oversized, innervated taste buds in *ShhCreER*^*T2*^*/Shh*^*f*^ mutant tongues. (**A-N**) Analyses of tongues from control and *ShhCreER*^*T2*^*/Shh*^*f*^ mutant embryos first exposed to tamoxifen at E11.5. (**A,B**) *In situ* hybridization with a riboprobe targeting *Shh* exon2 (the deleted allele) in E13.5 control (A) and mutant (B) tongues showing severely decreased *Shh* expression (purple) in the mutant tongue. (**C,D**) *Gli1 in situ* hybridization (brown) in parasagittal sections of E14.5 control (C) and mutant (D) tongues showing severe downregulation of *Gli1* expression in the mutant tongue, except in a small domain (arrows in D). (**E**) RT-qPCR analysis for *Ptch1* relative to *Actb* (β-actin) in tongues from E13.5 controls (n = 9) and mutants (n = 9). Severely decreased *Ptch1* levels in the mutant tongues as compared to the controls (*P* = 0.0000; mean values ± SD). (**F,G**) E15.5 control (F) and mutant (G) tongues after *in situ* hybridization with a riboprobe targeting both deleted (exon2) and non-deleted (exon1) *Shh*-coding sequences (brown) showing overproduction of oversized *Shh*-expressing taste buds in the mutant tongue. (**H-L**) Immunostaining (dark purple) for Keratin 8 (K8; H-J) and SOX2 (K,L) in parasagittal sections of E18.5 (H-J) and E16 (K,L) control (H,K) and mutant (I,J,L) tongues. The insets in (H) and (I) are enlarged images of the boxed areas in (H) and (I), respectively. The mutant tongues exhibit crowded and oversized taste buds (TB; I,L) and a fungiform papilla (FuP) abnormally harboring three TBs (J). Artefact (asterisk in I). (**M,N**) Parasagittal sections of E18 control (M) and mutant (N) tongues after K8 (green) and P2X2 (red) double staining showing innervated TBs. CvP, circumvallate papilla; T, tongue. Scale bars: 500 μm (A,B,F,G,H,I), 200 μm (C,D), 50 μm (J-L), and 25μm (M,N).

Remarkably, in *ShhCreER*^*T2*^*/Shh*^*f*^ mutants first exposed to TAM at E11.5, the tongues grew normally (Fig 5F–5I) despite severely reduced SHH signaling as revealed by *in situ* hybridization with probes targeting *Shh* exon2 (Fig 5A and 5B) [40] and *Gli1* (Fig 5C and 5D) as well as by *Ptch1* RT-qPCR (Fig 5E). *In situ* hybridization for *Shh*-exon1/exon2 (Fig 5F and 5G) and immunostaining for K8 and SOX2 (Fig 5H–5N) revealed that the E11.5 TAM-induced *ShhCreER*^*T2*^*/Shh*^*f*^ tongues overproduced fungiform papillae harboring oversized TBs. Quantification in E15.5 control and *ShhCreER*^*T2*^*/Shh*^*f*^ mutant tongues showed dramatically increased numbers of *Shh*+ spots (FPs and/or TBs) in the mutants (150 ± 6 in mutants *vs* 112 ± 4 in controls; mean ± SD; *P* = 0.000). Furthermore, quantification of K8+ cell clusters (TBs or parts of TBs) within fungiform papillae at E18.5 revealed a higher number of K8+ cell clusters in *ShhCreER*^*T2*^*/Shh*^*f*^ mutants when compared to controls (3.50 ± 0.50 K8+ clusters/section in mutants *vs* 1.43 ± 0.23 K8+ clusters/section in controls; mean ± SD; *P*<0.02). However, the mutant tongues assessed by K8 immuostaining were free from glandular and Merkel cell metaplasiae (Fig 5I and 5J; S2 Table). Similar to TBs in the E10.5-induced *ShhCreER*^*T2*^*/Shh*^*f*^ tongues, TBs within fungiform papillae in the E11.5 TAM-induced *ShhCreER*^*T2*^*/Shh*^*f*^ tongues were innervated by P2X2+ axons (Fig 5M and 5N; S2 Table). Thus, unlike in the central nervous system where SHH can act as an axonal chemoattractant [41], SHH is not required for local guidance of gustatory neurites towards fungiform papillae.

To further dissect the role of SHH in lingual cell fate specification, we analysed *Wnt1-CRE/Smo*^*f/f*^ mutants lacking SHH signaling specifically in the LM. As shown previously [6], the *Wnt1-CRE/Smo*^*f/f*^ tongues were abnormally small and cleft, and we found that they developed TBs and were free from glandular metaplasia (S2A–S2D Fig). The overlapping tongue anomalies in the E10.5 TAM-induced *ShhCreER*^*T2*^*/Shh*^*f*^ mutants and the *Wnt1-CRE/Smo*^*f/f*^ mutants demonstrate that SHH activity in the LM is necessary for tongue growth and morphogenesis but not for TB induction. The lack of glandular metaplasia in the oral tongue of the *Wnt1-CRE/Smo*^*f/f*^ mutants as well as in the E10.5 and E11.5 TAM-induced *ShhCreER*^*T2*^*/Shh*^*f*^ mutants, as compared to the *ShhGFPCRE/Smo*^*f/f*^ mutants, suggests the requirement for SHH-dependent mesenchymal inputs for ectopic gland formation. This situation is akin to that of the skin where induction of glandular metaplasia was found to require SHH signaling in the mesenchyme [19].

We also found that proper tongue development necessitates SHH inputs during a restricted period since the *ShhCreER*^*T2*^*/Shh*^*f*^ mutants and the *ShhCreER*^*T2*^*/Smo*^*f/f*^ mutants developed normal tongues following an initial TAM exposure at E12.5 (S2E–S2H’ Fig). That loss of SMO in the interplacodal epithelium of *K14-CRE/Smo*^*f/f*^ mutant tongues caused no tongue defects (S2I–S2J’ Fig) further demonstrates a temporal requirement for SHH signaling in tongue development. *K14-CRE* activity in the LE is late onset (≈ E13.5) (S2K–S2N Fig) [42], and taste placodes as well as TBs do not express K14 [29]. Together, these findings point to a restricted period before E12.5 during which loss of SHH signaling induces tongue defects and support previous findings in rat tongue organ cultures [15].

### *De novo* taste placode formation upon loss of SHH signaling

**Fig 6:**
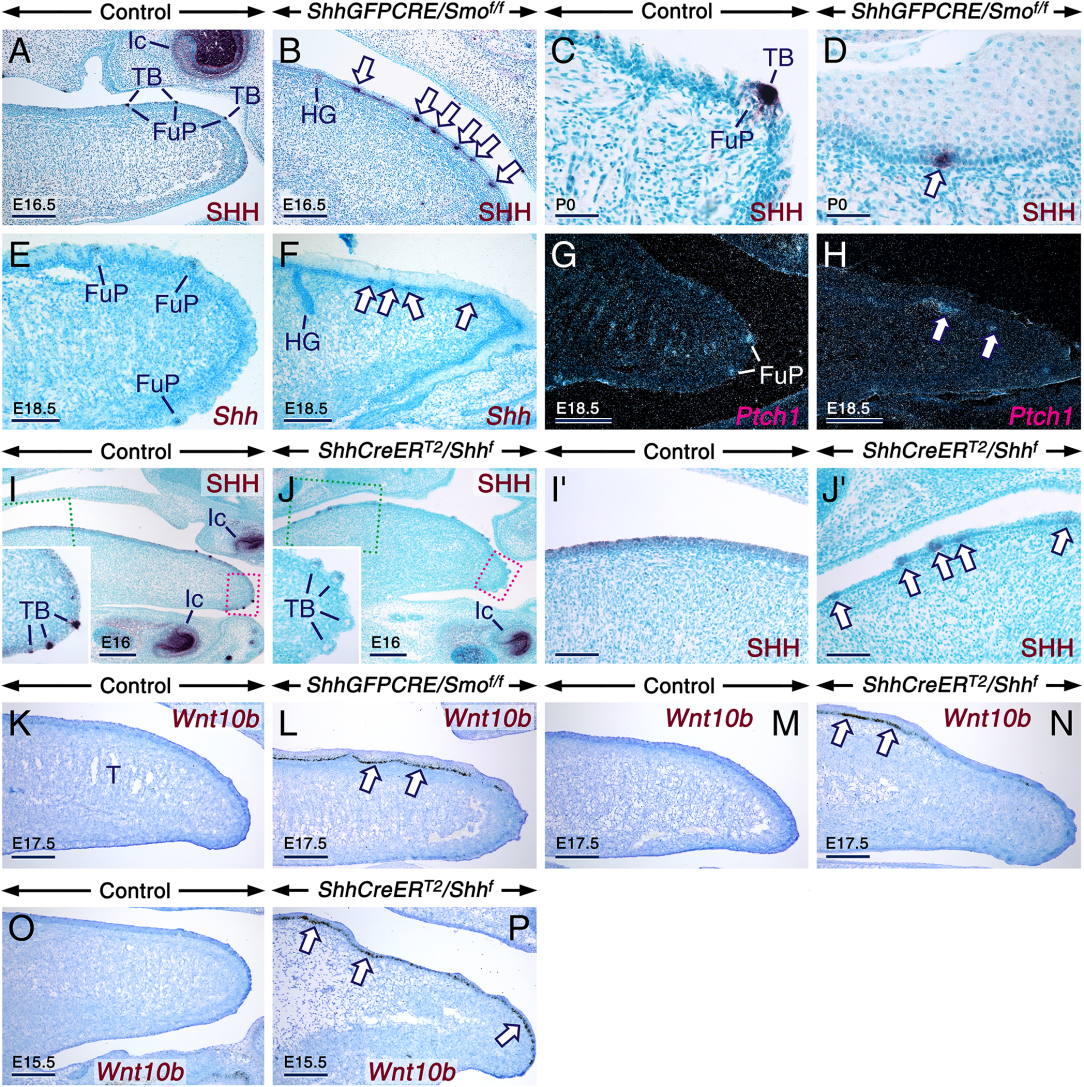
Ectopic and anachronic expression of *Shh* and *Wnt10b* in the lingual epithelium of *ShhGFPCRE/Smo*^*f/f*^ and tamoxifen-induced *ShhCreER*^*T2*^*/Shh*^*f*^ mutants specify *de novo* taste placode formation. (**A-D**) Anti-Sonic hedgehog-stained (SHH; dark purple) parasagittal tongue sections from controls (A,C) and *ShhGFPCRE/Smo*^*f/f*^ mutants (B,D) at E16.5 (A,B) and postnatal day P0 (C,D). (**E-H**) *Shh* (E,F) and *Ptch1* (G,H) *in situ* hybridization in parasagittal tongue sections from E18.5 control (E,G) and *ShhGFPCRE/Smo*^*f/f*^ (F,H) embryos. The signals appear as black or shiny dots in bright-field (E,F) or dark-field (G,H) images, respectively. SHH protein and *Shh* mRNA (SHH/*Shh*) are confined to taste buds (TB) of fungiform papillae (FuP) in the control tongues (A,C,E). The *ShhGFPCRE/Smo*^*f/f*^ tongues display SHH+/*Shh+* placode-like entities (arrows in B,D and F) underlain by a *Ptch1*+ mesenchyme (arrows in H). (**I-J’**) Anti-SHH-stained parasagittal tongue sections from E16 control (I) and *ShhCreER*^*T2*^*/Shh*^*f*^ mutant (J) embryos first exposed to tamoxifen at E11.5. (I'), (J'), and insets in (I) and (J) are enlarged images of the boxed areas in (I) and (J), respectively. TBs within fungiform papillae of the mutant tongue (inset in J) exhibit weaker SHH staining than those of the control tongue (inset in I). The mutant tongue shows SHH staining in placode-like structures in the epithelial basal layer (arrows in J’). (**K-P**) *Wnt10b in situ* hybridization (black) in parasagittal tongue sections. Sections from 17.5 control (K) and *ShhGFPCRE/Smo*^*f/f*^ mutant (L) embryos. Sections from 17.5 control (M) and *ShhCreER*^*T2*^*/Shh*^*f*^ mutant (N) embryos first exposed to tamoxifen (TAM) at E11.5. Sections from E15.5 control (O) and *ShhCreER*^*T2*^*/Shh*^*f*^ mutant (P) embryos first exposed to TAM at E10.5. All the mutant tongues show abnormal expression of *Wnt10b* in the epithelial basal layer (arrows) separated by *Wnt10b*-negative gaps. Ic, incisor tooth. Scale bars: 250 μm (I,J), 200 μm (A,B,E-H,K-P), 100 μm (I';J'), and 50 μm (C,D).

The active phase of FP patterning during which FPs are induced normally occurs between E12.5 and E14, and the FPs subsequently form TBs. Strikingly, from E15.5 onwards, unlike in controls, the *ShhGFPCRE/Smo*^*f/f*^ tongues consistently displayed SHH/*Shh*-expressing placode-like structures underlain by a *Ptch1*+ mesenchyme, suggesting *de novo* formation of FPs well after their induction normally has ceased (Fig 6A–6H). Basally located SHH+ cell clusters were also detected in the E11.5 TAM-induced *ShhCreER*^*T2*^*/Shh*^*f*^ mutant tongues. They displayed weaker SHH immunoreactivity than TBs in controls, likely subsequent to *Shh* gene expression ablation shortly after they express *Shh* (Fig 6I–6J’).

We next used a probe targeting *Wnt10b* which, like *Shh*, marks taste placodes [4]. In contrast to control tongues, which were devoid of *Wnt10b* transcripts after E14.5, the *ShhGFPCRE/Smo*^*f/f*^ as well as the E10.5 and E11.5 TAM-induced *ShhCreER*^*T2*^*/Shh*^*f*^ tongues abnormally exhibited *Wnt10b* expression separated by *Wnt10b*(–) regions within the basal layer of the LE (Fig 6K–6P). That the basally located placodes expressing *Shh* mRNA and protein (*Shh*/SHH) and *Wnt10b* were overlain by *Shh*/SHH(–) and *Wnt10b*(–) suprabasal epithelial cells (Fig 6A–6P) supports *de novo* induction of FPs. These data show sustained formation of taste placodes upon loss of SHH signaling, at least up to birth, and are reminiscent of the finding that *K14-CRE/Smo*^*f/f*^ mutants undergo *de novo* hair follicle formation [19]. Since *de novo* taste placode induction occurred in the *ShhGFPCRE/Smo*^*f/f*^ and TAM-induced *ShhCreER*^*T2*^*/Shh*^*f*^ mutant tongues, it could be concluded that loss of SHH signaling in the LE is the main cause of this phenomenon.

Our work here utilized a genetic approach to reveal that upon loss of SHH signaling, the mutant tongues transcended the time when FP induction normally ceases (E14.5) and formed *de novo* taste placodes. This phenomenon was not observed in previous studies using rodent tongue organ cultures, since in those studies the assessment of the impact of loss of SHH signaling on taste placode formation was limited to the active phase of FP patterning [12–15]. Furthermore, our findings that *in vivo* SHH inhibits FP induction during the active period of FP patterning not only concur with prior work *in vitro* [12–15], but they also demonstrate that SHH signaling is required to prevent sustained taste placode induction and that SHH operates strictly within the LE to fulfill these functions, without inputs from the LM. Similarly, genetic studies have demonstrated that Wnt/β-catenin signaling within the LE is crucial for FP induction [1]. Loss of Wnt/β-catenin activity downregulates *Shh* in FPs and *Ptch1* in FPs and their associated mesenchyme, whereas activation of Wnt/β-catenin signaling engenders expansion of *Shh* expression [14,43]. Moreover, findings from tongue organ cultures suggest that SHH inhibits Wnt/β-catenin activation, whereas Wnt/β-catenin signaling induces its own inhibitor, *i*.*e*. SHH [14]. However, how SHH impinges upon Wnt/β-catenin activity is unknown. Our findings of ectopic and anachronic induction of *Wnt10b* expression in mutants lacking SHH inputs suggest that SHH antagonizes Wnt/β-catenin signaling at least in part through inhibition of *Wnt10b* expression. Further supporting this notion is the finding that SHH inhibits *Wnt10b* expression in cultured teeth and whisker pads [44].

### Loss of *Shh* signaling during tongue development enhances retinoic acid signaling

The *ShhGFPCRE/Smo*^*f/f*^ tongues not only underwent glandular metaplasia, but they also formed von Ebner’s glands displaying mucous metaplasia. We postulated that the dramatic tongue anomalies caused by disrupted SHH signaling were a result of enhanced retinoic acid (RA) signaling. Components of the RA pathway are expressed during tongue development [45–50], and it is well-established that excess RA or vitamin A (a RA precursor) induces glandular/mucous metaplasia of various epithelia [51–56]. Strikingly, rodent embryos overexposed to RA/vitamin A exhibit tongue anomalies phenocopying those occurring in the *Shh*^*n/n*^ [57] and the various mutants described in this work, including aglossia, microglossia, bifid tongue, and adhesion of the LE to epithelia of the oral mucosa [58–61].

**Fig 7:**
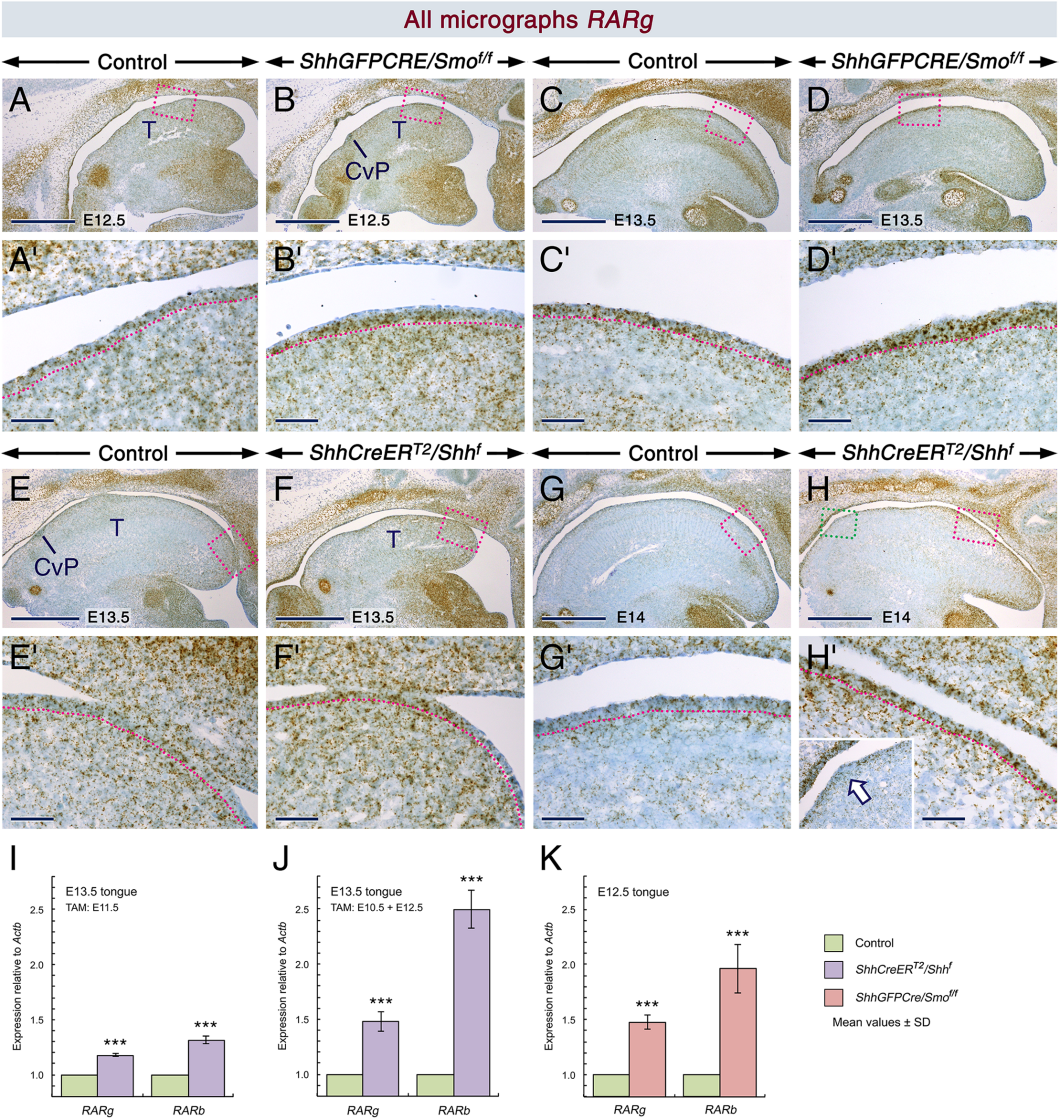
Upregulation of *RARb* and *RARg* expression in *ShhGFPCRE/Smo*^*f/f*^ and *ShhCreER*^*T2*^*/Shh*^*f*^ mutant tongues, indicating enhanced retinoic acid signaling. (**A-H’**) *RARg in situ* hybridization with oligonucleotide probes (brown) in parasagittal tongue sections. The dotted lines highlight the junction between the lingual epithelium and lingual mesenchyme. (**A-D’**) Sections from E12.5 (A,B) and E13.5 (C,D) control (A,C) and *ShhGFPCRE/Smo*^*f/f*^ mutant (B,D) embryos. (A'-D') are enlarged images of the boxed areas in (A-D). (**E-F’**) Sections from E13.5 control (E) and *ShhCreER*^*T2*^*/Shh*^*f*^ mutant (F) embryos first exposed to tamoxifen (TAM) at E10.5. (E’) and (F’) are enlarged images of the boxed areas in (E) and (F), respectively. (**G-H’**). Sections from E14 control (G) and *ShhCreER*^*T2*^*/Shh*^*f*^ mutant (H) embryos first exposed to TAM at E11.5. (G’) and (H’) are enlarged images of the boxed (red) areas in (G) and (H), respectively. The inset in (H’) is an enlarged image of the boxed (green) area in (H). The *RARg* hybridization signals in the lingual epithelium (LE) of the controls, to a large extent, appear as punctae, whereas in the LE of the mutants they appear as globular aggregates, indicating higher levels of *RARg* expression in the mutants relative to the controls. Epithelial foci in the mutants with weaker *RARg* signals than the rest of the LE (arrow in H’). (**I-K**) RT-qPCR analysis for *RARb* and *RARg* relative to *Actb* (β-actin). (I) Upregulation of *RARb* (*P* = 0.0000) and *RARg* (*P* = 0.0000) levels in tongues from E13.5 *ShhCreER*^*T2*^*/Shh*^*f*^ mutants (n = 6) as compared to tongues from controls (n **=** 6) first exposed to TAM at E11.5. (J) Upregulated *RARb* (*P* = 0.0000) and *RARg* (*P* = 0.0003) levels in tongues from E13.5 *ShhCreER*^*T2*^*/Shh*^*f*^ mutants (n = 6) as compared to tongues from controls (n = 6) first exposed to TAM at E10.5. (K) Upregulation of *RARb* (*P* = 0.0015) and *RARg* (*P* = 0.0000) levels in tongues from E12.5 *ShhGFPCRE/Smo*^*f/f*^ mutants (n = 6) as compared to tongues from controls (n = 6). The data are mean values ± SD. CvP, circumvallate papilla; T, tongue. Scale bars: 500 μm (A-H) and 50 μm (A’-H’).

To test our hypothesis, we first assessed the expression of *RARb* and *RARg*, well-established transcriptional targets of RA signaling that encode the nuclear retinoic acid receptors RARβ and RARγ, respectively [62]. We found that relative to controls, the LE of the *ShhGFPCRE/Smo*^*f/f*^ mutants (Figs 7A–7D’ and S3A–S3L) as well as that of the E10.5 (Figs 7E–7F’ and S3M–S3P) and E11.5 (Fig 7G–7H’) TAM-induced *ShhCreER*^*T2*^*/Shh*^*f*^ mutants displayed stronger *RARb* and/or *RARg* hybridization signals. Furthermore, RT-qPCR revealed significant upregulation of *RARb* and *RARg* expression in the mutants (Fig 7I–7K).

Consistent with these findings, anti-RARγ staining revealed that the *ShhGFPCRE/Smo*^*f/f*^ tongues exhibited stronger RARγ immunolabelling in the LE than that of controls from E11.5 to E14.5 (S4A–S4G Fig) except in epithelial foci, which could be developing squamous hyperplastic sites (S4G Fig). Noticeably, RARγ was readily detectable in TBs which expressed *RARg* transcripts (S4E and S4H–S4K Fig), and the lingual glands were also RARγ+ (S4L–S4N Fig). These data show that the LE and its derivatives produce RARγ protein and that RA signaling is enhanced in the LE upon loss of SHH inputs.

### Enhanced RA signaling upon loss of SHH signaling during tongue development is due to loss of SHH-dependent *Cyp26a1/c1* expression

Transcripts encoding the RA synthesizing enzymes RALDH1-3 have been shown to be expressed in the LE at E14.5 and E16.5 [49]. To assess the distribution patterns of RALDH1-3 during tongue development and to determine whether they are altered in the *ShhGFPCRE/Smo*^*f/f*^ tongues, we immunostained sections from controls and mutants from E11.5 onwards. We found that RALDH1-3 were expressed in the LE from E11.5 onwards as well as in the orthotopic and heterotopic lingual glands (S5 and S6 Figs). We also found that FPs were RALDH3+ (S5Q and S5R Fig), whereas TBs were RALDH1+ (S5S–S5T and S5Y–S5Z Fig). Thus, the LE and its derivatives in controls and mutants are exposed to RALDH-derived RA. However, besides abnormal enhancement of RALDH3 and RALDH2 staining in the LE of the mutants at E11.5 (S5F Fig) and E16.5 (S5V Fig), respectively, there were no major alterations in RALDH1-3 distribution patterns in the mutants.

**Fig 8:**
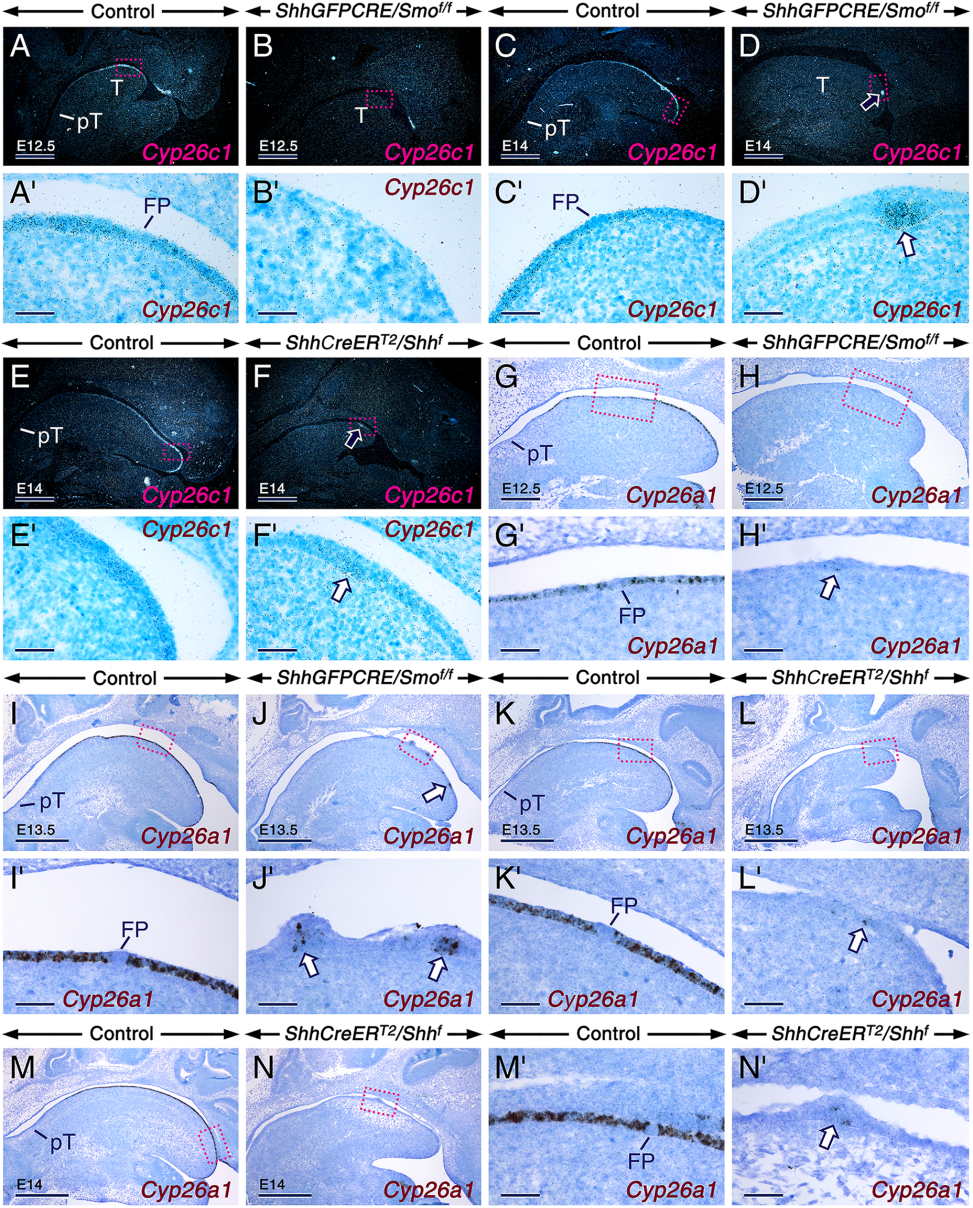
Dramatic downregulation of *Cyp26a1* and *Cyp26c1* expression in the lingual epithelium of *ShhGFPCRE/Smo*^*f/f*^ and *ShhCreER*^*T2*^*/Shh*^*f*^ mutant tongues. (**A-N’**) Parasagittal tongue (T) sections from controls and mutants after *in situ* hybridization with a ^35^S-UTP-labelled riboprobe for *Cyp26c1* (A-F’) and oligonucleotide probes for *Cyp26a1* (G-N’; black). Genotypes are indicated in the panels. (**A-D**) Dark-field images showing *Cyp26c1* expression (shiny dots) in E12.5 (A,B) and E14 (C,D) control and *ShhGFPCRE/Smo*^*f/f*^ mutant tongues. (**A’-D’**) Enlarged bright-field images (hybridization signals appear as black dots) of the boxed areas in (A-D). (**E,F**) Dark-field images showing *Cyp26c1* expression in tongue sections from E14 control and *ShhCreER*^*T2*^*/Shh*^*f*^ mutant embryos first exposed to tamoxifen (TAM) at E10.5. (E’) and (F’) are enlarged, bright-field images of the boxed areas in (E) and (F), respectively. All mutants show virtual abrogation of *Cyp26c1* expression (B,D,F), except in epithelial foci (arrows in D,D’,F and F’). Absence of *Cyp26c1* transcripts in fungiform placodes (A’,C’) and pharyngeal tongue (A,C,E) in controls. (**G-J’**) *Cyp26a1* expression in tongue sections from E12.5 (G,H) and E13.5 (I,J) control and *ShhGFPCRE/Smo*^*f/f*^ mutant embryos. (G’-J’) Enlarged images of the boxed areas in (G-J). (**K**-**L’**) *Cyp26a1* expression in tongue sections from E13.5 control and *ShhCreER*^*T2*^*/Shh*^*f*^ mutant embryos first exposed to TAM at E10.5. (K’) and (L’) are enlarged images of the boxed areas in (K) and (L), respectively. (**M-N’**) *Cyp26a1* expression in tongue sections from E14 control and *ShhCreER*^*T2*^*/Shh*^*f*^ mutant embryos first exposed to TAM at E11.5. (M’) and (N’) are enlarged images of the boxed areas in (M) and (N), respectively. All mutants show virtual absence of *Cyp26a1* expression, except in epithelial foci (arrows in H’,J,J’,L’ and N’). *Cyp26a1* transcripts are excluded from fungiform placodes (G’,I’,K’ and M’) and pharyngeal tongue (G,I,K,M) in the controls. FP, fungiform placode; PT, pharyngeal tongue. Scale bars: 500 μm (A-F,I-N), 200 μm (G,H), and 50 μm (A’-N’).

**Fig 9:**
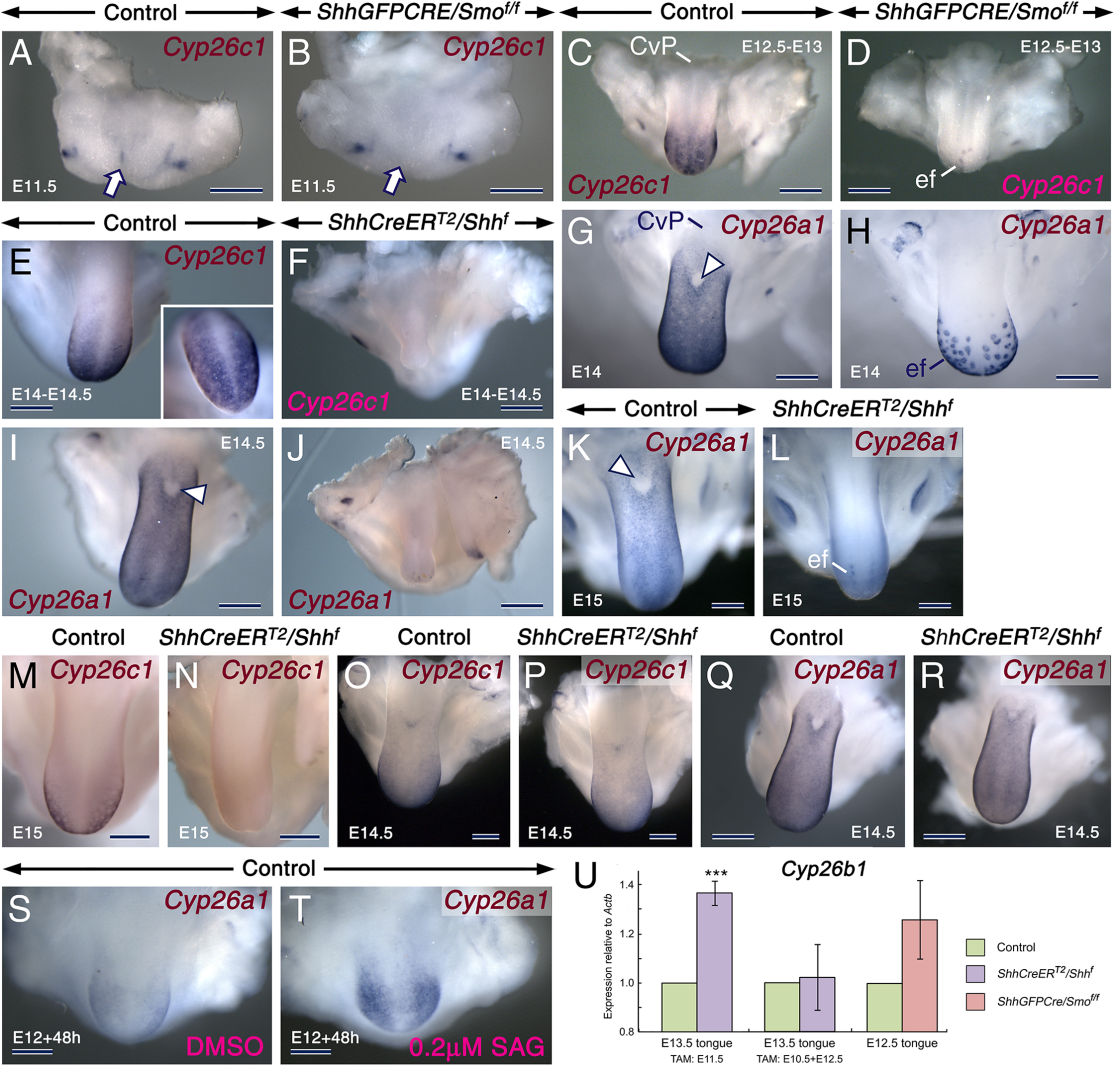
SHH signaling is required for maintenance/reinforcement but not for induction of *Cyp26a1* and *Cyp26c1* expression. (**A-R**) *In situ* hybridization for *Cyp26c1* and *Cyp26a1* in the developing tongue (dark blue/brown). (**A**,**B**) E11.5 control (A) and *ShhGFPCRE/Smo*^*f/f*^ mutant (B) tongues showing *Cyp26c1* expression in the control tongue (arrow in A) and absence of *Cyp26c1* expression in the mutant tongue (arrow in B). (**C**,**D**) *Cyp26c1* expression in E12.5-E13 control (C) and *ShhGFPCRE/Smo*^*f/f*^ mutant (D) tongues. (**E**,**F**) *Cyp26c1* expression in tongues from E14-E14.5 control (E) and *ShhCreER*^*T2*^*/Shh*^*f*^ mutant (F) embryos first exposed to tamoxifen (TAM) at E10.5. The inset in (E) is an enlarged image of the specimen in (E). (**G,H**) *Cyp26a1* expression in E14 control (G) and *ShhGFPCRE/Smo*^*f/f*^ mutant (H) tongues. (**I,J**) *Cyp26a1* expression in tongues from E14.5 control (I) and *ShhCreER*^*T2*^*/Shh*^*f*^ mutant (J) embryos first exposed to TAM at E10.5. (**K-N**) *Cyp26a1* (K,L) and *Cyp26c1* (M,N) expression in tongues from E15 control (K,M) and *ShhCreER*^*T2*^*/Shh*^*f*^ mutant (L,N) embryos first exposed to TAM at E11.5. Severe downregulation of *Cyp26a1/Cyp26c1* expression in all mutants, except in epithelial foci (ef). Fungiform placodes or taste buds, which appear as unstained spots (C,G, inset in E), and the circumvallate papilla (CvP) are *Cyp26a1/Cyp26c1*-negative. Artefact due to loss of the thin epithelium of the intermolar eminence during tissue processing (arrowheads in G, I and K). (**O-R**) Unaltered *Cyp26c1* (O,P) and *Cyp26a1* (Q,R) expression in tongues from E14.5 control (O,Q) and *ShhCreER*^*T2*^*/Shh*^*f*^ mutant (P,R) embryos first exposed to TAM at E12.5. (**S,T**) *Cyp26a1* expression in tongue explants from E12 control embryos after *in vitro* culture for 2 days with DMSO (S) and 200 nM SAG (T). SAG enhanced *Cyp26a1* hybridization signals in the anterior tongue (blue) but failed to induce ectopic *Cyp26a1* expression in the posterior tongue and mandibular structures. (**U**) RT-qPCR analysis for *Cyp26b1* relative to *Actb* (β-actin) in tongues from E12.5 controls (n = 6) and *ShhGFPCRE/Smo*^*f/f*^ mutants (n = 6), in tongues from E13.5 controls (n = 6) and *ShhCreER*^*T2*^*/Shh*^*f*^ mutants (n = 6) first exposed to TAM at E10.5, and in tongues from E13.5 controls (n = 8) and *ShhCreER*^*T2*^*/Shh*^*f*^ mutants (n = 8) first exposed to TAM at E11.5. *Cyp26b1* levels are upregulated in mutants relative to controls first exposed to TAM at E11.5 (*P* = 0.0000). The data are mean values ± SD. Scale bars: 500 μm (A-N,Q,R) and 300 μm (O,P,S,T).

RA signaling is tightly regulated by activities of RALDH1-3 and the RA catabolic enzymes belonging to the CYP26 family [63,64]. As the *ShhGFPCRE/Smo*^*f/f*^ mutant tongues had no major alterations in RALDH distribution, we reasoned that the increased RA signaling could be caused by decreased production of CYP26A1 and CYP26C1 which are expressed in the LE [65–67]. We found that *Cyp26a1/c1* transcripts were dramatically reduced in developing tongues dissected from *ShhGFPCRE/Smo*^*f/f*^ mutants as well as from E10.5 and E11.5 TAM-induced *ShhCreER*^*T2*^*/Shh*^*f*^ mutants compared to controls (Fig 8 and Fig 9). However, some *Cyp26a1*/*c1*-expressing epithelial foci persisted in the mutants (Fig 8 and Fig 9). These are likely developing focal squamous hyperplasiae as shown in tissue sections (arrows in Fig 8), which consistently develop in the *ShhGFPCRE/Smo*^*f/f*^ and TAM-induced *ShhCreER*^*T2*^*/Shh* ^*f*^ mutants. Strikingly, we found that during the active phase of FP patterning, *Cyp26a1*/*c1* were excluded from FPs (Fig 8 and Fig 9). When FP induction is complete at E14.5 *Cyp26a1/c1* levels started to diminish (Fig 9I, 9K and 9M), and after E16.5, *Cyp26a1/c1* were no longer expressed in the LE [65,66]. Furthermore, *Cyp26a1*/*c1* expression was excluded from the LE at the level of the developing circumvallate papilla and from the pharyngeal tongue (Fig 8 and Fig 9). *Cyp26b1*, another CYP26 family member, is expressed exclusively in the LM [65]. We found that *Cyp26b1* levels (Fig 9U) in the mutant tongues were either essentially unaltered (*ShhGFPCRE/Smo*^*f/f*^ and E10.5 TAM-induced *ShhCreER*^*T2*^*/Shh*^*f*^) or upregulated (E11.5 TAM-induced *ShhCreER*^*T2*^*/Shh*^*f*^), confirming enhanced *RARb* and *RARg* levels in the LE of the mutants. Unlike in both the *ShhGFPCRE/Smo*^*f/f*^ and in the E10.5 and E11.5 TAM-induced *ShhCreER*^*T2*^*/Shh*^*f*^ mutant tongues, *Cyp26a1*/*c1* levels were unaltered in E12.5 TAM-induced *ShhCreER*^*T2*^*/Shh*^*f*^ mutant tongues (Fig 9O–9R). Thus, a critical temporal window of SHH activity is necessary for expression of *Cyp26a1/c1* in the LE. These data, together with our findings indicating a temporal window during which loss of SHH signaling causes FP mispatterning, suggest that early loss (before E12.5) of SHH signals is necessary for downregulation of *Cyp26a1/c1* expression in the LE during FP patterning (E12.5-E14) and for the entailing patterning defects.

While our data indicate that *Cyp26a1*/*c1* expression in the LE requires SHH inputs, it is unlikely that the transcripts are direct targets of SHH signaling. At E11.5, despite the presence of significant SHH activity in the tongue primordium [6,8], only weak *Cyp26a1/c1* signals were detectable (Fig 9A) [67]. Furthermore, treatment of tongue/mandible explants with SAG, a SMO agonist, enhanced *Cyp26a1* signals in the LE but failed to induce ectopic *Cyp26a1* expression in epithelia of the pharyngeal tongue and mandible (Fig 9S and 9T). These observations suggest that SHH signaling is required for maintenance and/or reinforcement of *Cyp26a1*/*c1* expression in the LE but not for their initial induction.

The virtual absence of *Cyp26a1/c1* transcripts in taste placodes and TBs amid high expression levels in the interplacodal LE is a conundrum in view of the data indicating the requirement of SHH signaling for *Cyp26a1/c1* expression. However, taste placodes and TBs, similar to other SHH-producing signaling centers including the notochord, neural floor plate and tooth enamel knots [18,68,69], did not exhibit higher levels of *Ptch1* and *Gli1* than the nearby *Shh*-negative-SHH-responsive tissues. Furthermore, *Gli1-lacZ* mice revealed that *Shh*-expressing cells in taste placodes and papillae are less SHH-responsive than the neighbouring epithelial and mesenchymal cells [70]. Moreover, taste placodes, the notochord, and tooth enamel knots are induced after loss of *Shh* or *Smo* [our present data; 9,18,40,71]. In light of these observations, we propose that SHH signaling is attenuated in taste placodes and TBs and that this, combined with inputs from yet to be identified factor(s), leads to absence of *Cyp26a1/c1* in these structures.

### RA signaling is required for orthotopic lingual gland formation, and enhanced RA activity causes glandular but not Merkel cell metaplasiae in the SMO-deficient LE

That RA signaling is activated upon loss of SHH signaling strongly suggests that the glandular metaplasia, *i*.*e*. cell fate switch prompting the LE in the *ShhGFPCRE/Smo*^*f/f*^ oral tongue to form heterotopic glands, is a direct consequence of hyperactivation of RA signaling. Indeed, it is well-established that excess RA and Vitamin A induces glandular/mucous metaplasia of various epithelia [51–56]. To functionally test this scenario, we cultured E11.5 tongues and the surrounding mandibular structures in the presence of retinoids and inhibitors of RA signaling. We found that treating explants from control embryos with retinoids, including *all-trans* RA, CD1530 (RARγ selective agonist) and CD2314 (RARβ selective agonist) induced formation of K8+ heterotopic glands in the oral tongue, mimicking the *ShhGFPCRE/Smo*^*f/f*^ *in vivo* phenotype (S7A–S7E Fig; S3 Table). *ShhGFPCRE/Smo*^*f/f*^ tongue explants treated with vehicle (DMSO) recapitulated the *in vivo* defects, including glandular and Merkel cell metaplasiae as well as formation of K8(–) epithelial foci (S7F and S7I Fig; S3 Table). Importantly, exposure to BMS493 (pan-RAR antagonist) and DEAB (RA synthesis inhibitor) inhibited heterotopic gland formation in the *ShhGFPCRE/Smo*^*f/f*^ explants (S7F–S7K Fig; S3 Table). Neither BMS493 nor DEAB inhibited lingual Merkel cell metaplasia in the mutant explants (S7H and S7J Fig). Thus, lingual Merkel cell metaplasia is not caused by enhanced RA signaling. Furthermore, BMS493 and DEAB failed to inhibit development of the K8(–) foci in the mutants (S7H and S7J Fig), consistent with our findings suggesting that these are subjected to low RA signaling owing to persistent *Cyp26a1/c1* expression. To determine whether RA signaling is required for development of von Ebner’s glands and posterior lingual glands, which normally develop in *Cyp26a1/c1*-free domains, we treated E12.5 control tongues/mandibles with DMSO or BMS493 (S3 Table). While the DMSO-treated explants formed K8+ von Ebners’ glands and K8+ posterior lingual glands, the BMS493-treated explants failed to develop von Ebners’ glands and displayed developmentally arrested posterior lingual glands (S7L–S7M’ Fig; S3 Table). These data demonstrate that abnormal RA activation in the *ShhGFPCRE/Smo*^*f/f*^ tongues causes glandular metaplasia and that development of von Ebners’ glands and posterior lingual glands requires RA inputs. They also show that the LE responds to RA. Despite this evidence and well-established expression of components of the RA pathway during tongue development, various transgenic mice reporting RA activity failed to show transgene activity in the LE and its derivatives [72–75]. Likewise, we were unable to detect RA activity in the LE except in subsets of desquamating cells of controls and *ShhGFPCRE/Smo*^*f/f*^ mutants carrying the *RARE-hsp68-LacZ* transgene, another reporter of RA activity [76] (S8A–S8F Fig). Moreover, retinoid-treated tongue explants from *RARE-hsp68-LacZ* embryos failed to undergo epithelial *RARE-hsp68-LacZ* activation, whereas tails from these embryos displayed transgene activity (S8G–S8I Fig). These observations and findings suggest that all the above transgenes are inactive in the LE and its derivatives. Accordingly, several studies pointed to lack of accuracy of such transgenic mice for visualization of RA activity in various tissues [77–79].

### RA signaling promotes fungiform placode formation

**Fig 10:**
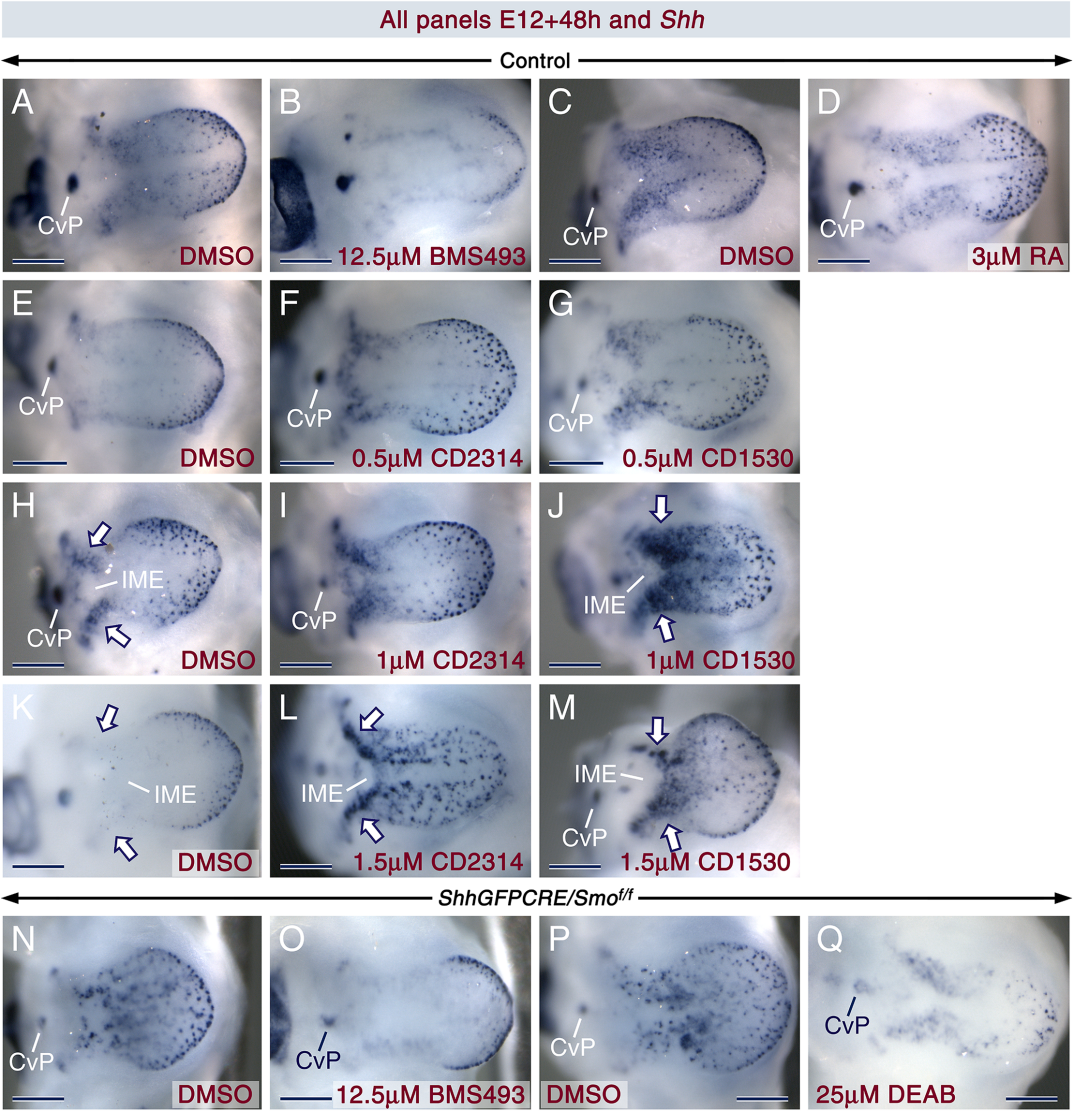
Retinoids induce formation of oversized fungiform placodes, whereas inhibitors of RA signaling impinge upon their development. (**A-Q**) *Shh in situ* hybridization (dark blue) in E12 tongue explants from control (without the *CRE* and/or the *Smo* floxed alleles; A-M) and *ShhGFPCRE/Smo*^*f/f*^ mutant (N-Q) embryos after *in vitro* culture for 48h. The explants were treated with DMSO (control for each treatment/concentration), BMS493, DEAB and retinoids [*all trans-*retinoic acid (RA), CD2314 and CD1530]. (**A,B**) DMSO (A) and 12.5 μM BMS493 (B). The BMS493-treated tongue exhibit large areas devoid of *Shh* expression. (**C,D**) DMSO (C) and 3 μM RA (D). (**E-G**) DMSO (E), 0.5 μM CD2314 (F), and 0.5 μM CD1530 (G). (**H-J**) DMSO (H), 1 μM CD2314 (I), and 1 μM CD1530 (J). (**K-M**) DMSO (K), 1.5 μM CD2314 (L), and 1.5 μM CD1530 (M). The retinoids promoted the formation of relatively oversized *Shh*+ spots, likely fungiform placodes. Relative to DMSO (arrows in H and K), CD2314 at 1.5 μM as well as CD1530 at 1 μM and 1.5 μM, promoted development of oversized *Shh*+ spots in the areas flanking the presumptive intermolar eminence (arrows in J, L and M). (**N-Q**) Tongue explants from mutant embryos treated with DMSO (N,P), 12 μM BMS493 (O), and 25 μM DEAB (Q) showing large areas virtually devoid of *Shh* expression after BMS493 and DEAB treatment. CvP, circumvallate papilla; IME, presumptive intermolar eminence. Scale bars: 200 μm (A-Q).

We found that loss of SHH signaling elicits enhanced development of FPs amidst increased RA activity. This strongly suggests that RA signaling promotes FP formation. To test for direct evidence of the phenomenon, we treated tongue explants from control embryos with retinoids and found that they induced development of oversized *Shh*+ placodes (Fig 10C–10M; S4 Table). Furthermore, explants exposed to CD2314 and CD1530 exhibited ectopic *Shh* expression along the tongue midline and enhanced *Shh* signals, including in enlarged spots within two domains flanking the prospective intermolar eminence (Fig 10J, 10L and 10M). By contrast, explants from controls (Fig 10A and 10B; S4 Table) and *ShhGFPCRE/Smo*^*f/f*^ mutants (Fig 10N–10Q; S4 Table) exposed to BMS493 or DEAB exhibited large areas with weak or no *Shh* signals. Taken together with our findings of occurrence of RA signaling in taste placodes (expression of RALDHs amid absence of *Cyp26a1/c1*), these data show that RA promotes taste placode formation.

### Conclusions

In this study, we adopted *in vivo* genetic approaches and experimental manipulation in tongue organ cultures *in vitro* which enabled us to reveal a temporal requirement for SHH signaling for proper tongue development. Before E12.5, SHH signaling in the LE controls patterning and cell fate specification, whereas in the LM, SHH inputs are required to drive growth and morphogenesis (S9 Fig). We uncovered unanticipated relationships between SHH and RA signaling during patterning of the LE (S9 Fig). We discovered that genetic loss of SHH activity in the LE leads to enhanced RA signaling and that in the absence of SHH inputs, the LE overproduces oversized FPs and TBs and can even undergo *de novo* taste placode formation. We showed that *Cyp26a1/c1* expression in the interplacodal epithelium requires SHH inputs and that retinoids or RA signaling inhibitors induce or inhibit FP formation, respectively. These findings provide compelling evidence that SHH inhibits FP induction by abating RA activity in the interplacodal epithelium.

It is well-established that RA signaling plays a central role in controlling antero-posterior patterning of the limb, hindbrain, gut, and trunk [80–83]. Our genetic studies showed that loss of SHH signaling in the LE not only leads to formation of heterotopic glands in the oral tongue, but also causes overgrowth and mucous metaplasia of von Ebner’s glands. Furthermore, in tongue explants retinoids and RA inhibitors induced and inhibited lingual gland formation, respectively. These findings strongly suggest that antero-posterior patterning of the LE is controlled by antagonistic SHH and RA activities, where SHH inhibits and RA promotes lingual gland formation in the oral and pharyngeal tongues (S9 Fig). To our knowledge, neither *de novo* taste placode induction nor cell fate switch causing glandular and Merkel cell metaplasiae in the oral tongue, as in the *ShhGFPCRE/Smo*^*f/f*^ mutants, have been reported in mouse models or in experimental studies *in vitro*, suggesting that SHH and RA pathways play key roles in cell fate specification within the LE (S9 Fig). Our findings may provide insight into the etiopathogenesis of Merkel cell carcinoma of the tongue which remains an enigma [84].

We found that genetic loss of SHH signaling in the LE causes ectopic expression of *Shh* amidst abnormal activation of RA signaling and that retinoids induce expansion of *Shh* expression in tongue organ cultures. As normal levels of *Shh* expression in FPs require Wnt/β-catenin inputs [14,43], these observations raise the question as to the relationships between RA and Wnt/β-catenin signaling during FP patterning. The Hedgehog and RA pathways are crucial for development and homeostasis of a vast array of tissues and organs, and deregulated Hedgehog signaling generates neoplasia [10,11,81–83]. Our findings thus constitute a basis for future research aimed at deciphering the interactions between these pathways during development and disease, a largely unexplored topic.

## Materials and methods

### Ethics statement

The experiments using mice were reviewed and approved by the Animal Research Ethics Committee in Göteborg, Sweden (Dnr. 230–2010, 174–2013 and 40–2016). Mouse studies were also carried out under approved protocols in strict accordance with the policies and procedures established by the University of California, San Francisco (UCSF) Institutional Animal Care and Use Committees (UCSF protocol AN084146). Mice were euthanised by cervical dislocation.

### Mouse lines and tamoxifen induction of alleles

The different transgenic or knockin mice used in this work include the *K14-CRE* [40], *ShhGFPCRE* and *ShhCreER*^*T2*^ [85], *Wnt1-CRE* [6], and *R26R* [86] mice. We crossed *K14-CRE*, *ShhGFPCRE* or *Wnt1-CRE* males with females carrying the *Shh* or *Smo* floxed alleles (*Shh*^*f/f*^and *Smo*^*f/f*^) as described previously [17,18,40,87,88]. Thereafter *K14-CRE/Smo*^*+/f*^, *ShhGFPCRE/Smo*^*+/f*^, *ShhCreER*^*T2*^, *ShhCreER*^*T2*^*/Smo*^*+/f*^, or *Wnt1-CRE/Smo*^*+/f*^ males were crossed with *Shh*^*f/f*^ *or Smo*^*f/f*^ females to generate mutants. *K14-CRE*, or *ShhGFPCRE* males were crossed with *R26R* females to generate reporter embryos carrying the *CRE* and *R26R* alleles (*K14-CRE/R26R* and *ShhGFPCRE/R26R*). The *RARE-hsp68-LacZ* transgenic mice were as described [76]. We generated *ShhGFPCRE/Smo*^*+/f*^*/RARE-hsp68-LacZ* males which were thereafter crossed with *Smo*^*f/f*^ females to generate *ShhGFPCRE/Smo*^*f/f*^*/RARE-hsp68-LacZ* mutants and controls (without the *CRE* and/or the *Smo* floxed alleles). *ShhGFPCRE/Smo*^*+/f*^ (or without *CRE*) without the *RARE-hsp68-LacZ* transgene were used as negative controls. For tamoxifen (TAM) induction of the *ShhCreER*^*T2*^*/Shh*^*f*^ and *ShhCreER*^*T2*^*/Smo*^*f/f*^ mutants, pregnant females received intraperitoneal injections of TAM (dissolved in corn oil) every other day (excluding the day of embryo harvest) with the first injection consisting of 0.2 mg/g body weight (bw) TAM and the subsequent injections consisting of 0.1 mg/g bw. All mutants and their control littermates were identified by genotyping using DNA from the tip of the tail or a piece of embryonic tissue and/or, when possible, by their external phenotypes.

### Histology, immunostaining, *in situ* hybridization, *Lac-Z* staining and GFP imaging

The number of tongues from control and mutant embryos processed for *in vivo* studies is described in S1 Text. Heads or mandibles/tongues from the various mutants and their control littermates were prepared for sectioning or used as whole-mounts. For histological staining (alcian blue van Gieson), immunohistochemistry, and immunofluorescence, specimens were fixed in ethanol-acetic acid as previously described [89,90] or in 4% paraformaldehyde in 1X PBS (PFA/PBS). For *in situ* hybridization, specimens were fixed in 4% PFA/PBS at 4°C. The specimens were thereafter processed for whole-mount *in situ* hybridization or embedded in paraffin. Six-μm paraffin sections were used for immunostaining and *in situ* hybridization. For histology, immunostaining, and *in situ* hybridization the specimens were processed as previously described [18,19,89–91]. Sections were counterstained with Richardson’s azure II-methylene blue and methyl green after *in situ* hybridization and immunostaining, respectively. For green fluorescent protein imaging, tongues from *ShhGFPCRE/+* (controls) and *ShhGFPCRE/Smo*^*f/f*^ mutants were fixed overnight in 1% PFA/PBS at 4°C, rinsed in PBS, and imaged as whole-mounts. For visualization of *LacZ* activity, specimens were fixed overnight in 2% PFA/PBS at 4°C. Whole-mounts and cryostat tissue sections were subsequently processed for β-galactosidase histochemistry as previously described [19,90].

### Antibodies and probes

The following antibodies were obtained and used for immunostaining: the Troma-1 (anti-Keratin 8) rat monoclonal antibody (1:3000) developed by P. Brulet and R. Kemler from the DSHB developed under the auspices of the NICHD and maintained by the University of Iowa, Department of Biological Sciences, Iowa City, IA, USA; goat anti-ALDH1A2 (RALDH2, 1:400) and goat anti-SOX2 (1:3500) from Santa Cruz Biotechnology; rabbit anti-Keratin 6 (1:10,000) and chicken anti-Keratin 15 (1:10,000) from Covance; rabbit anti-Homer 1 (1:3000) and rabbit anti-Rab3c (1:1000) from Proteintech Group; rabbit Mab anti-ALDH1A1 (RALDH1, 1:400) and chicken anti-200 kD Neurofilament heavy (NF200; 1:4000) from Abcam; rabbit anti-ALDH1A3 (RALDH3, 1:400) from Sigma Life Science; rabbit anti-RARγ (1:3000) from LifeSpan Biosciences; goat anti-SHH (1:200) from R&D Systems; rabbit anti-P2X2 (1:200) from US Biological; and rabbit anti-SHH (Ab80; 1:800) [89,92]. Ab80 recognizes both SHH and Indian hedgehog (IHH) proteins [89,92], hence staining of craniofacial cartilage which produces IHH was used as internal control in sections from mutants lacking *Shh* gene function.

The following riboprobes were generated from linearized plasmids and used for *in situ* hybridization on tissue sections (^35^S-UTP-labelled riboprobes) and/or whole-mount *in situ* hybridization (Dig-labelled riboprobes): *Shh* targeting exon1 and exon2 [39; AP. McMahon, personal communication]; *Ptch1*, *Gli1*, and *Shh*/*exon2* [40]; *Cyp26a1* [93]; *RARg* and *RARb* [45]; and *Cyp26c1* [66]. Tissue sections were processed for *in situ* hybridization with oligonucleotide probes targeting Mm-*Gli1* (NM_010296.2; target sequences: 25–1205); Mm-*Cyp26a1* (NM_007811.2; target sequences: 303–1556), Mm-*Ptch1* (NM_008957.2; target sequences: 2260–3220), Mm-*RARg* (NM_007811.2; target sequences: 944–2623) and Mm-*Wnt10b* (NM_011718.2; target: 989–2133) using the RNAscope technology (Advanced Cell Diagnostics).

### Quantification of taste buds and ectopic Merkel cells

#### Quantification of *Shh*-expressing taste buds/taste placodes

The number of *Shh*-expressing taste buds/taste placodes was determined by counting *Shh*+ spots in micrographs of tongues processed for whole-mount *in situ* hybridization. Photoshop’s pencil tool was used to mark the spots counted. Quantification was performed in E15.5 control (n = 2 from the same litters as the mutants and n = 3 from other litters) and *ShhGFPCRE/Smo*^*f/f*^ mutant (n = 3) tongues, as well as in E15.5 control (n = 3) and *ShhCreER*^*T2*^*/Shh*^*f*^ mutant (n = 4) tongues from embryos first exposed to tamoxifen at E11.5. Student’s t-test was used for statistical analyses.

#### Quantification of Keratin 8-expressing (K8) taste buds and ectopic Merkel cells after double immunofluorescence for various markers

Ribbons consisting of 3–4 parasagittal sections of paraffin-embedded tongues were collected onto slides. Each slide held 3–4 ribbons of sections, and each ribbon on a given slide was taken every 234–240 μm. Taste buds (TBs) and ectopic Merkel cells were counted in one section per ribbon. All the other TBs and Merkel cells (different from those already counted) that were visible in the remaining sections were also included in the quantification. The number of K8+ solitary cells in the basal layer of the lingual epithelium (ectopic Merkel cells) and the number of K8+ TBs expressing Sonic Hedgehog (SHH), Homer1 and Rab3c were determined after K8/SHH, K8/Homer1 and K8/Rab3c immunostaining. The number of K8+ TBs and K8+ ectopic Merkel cells that were innervated by gustatory neurites was determined after K8/P2X2 immunostaining. The number of ectopic Merkel cells that were innervated by NF-200+ neurites was determined after K8/NF-200 immunostaining. The number of tongues from control and mutant embryos processed for immunofluorescence is described in S1 Table and S2 Table.

#### Quantification of K8-expressing cell clusters in fungiform papillae

Paraffin-embedded tongues from E18.5 control (n = 3) and *ShhCreER*^*T2*^*/Shh*^*f*^ mutant (n = 3) embryos first exposed to tamoxifen at E11.5 were sectioned in their entirety in sagittal/parasagittal planes. After immunohistochemistry for K8, K8+ cell clusters in fungiform papillae were counted in all serial sections and averaged per section. Student’s t-test was used for statistical analyses.

### *In vitro* explant cultures

Mandibular arches with the developing tongue were dissected from control embryos (without the *CRE* and/or the *Smo* floxed alleles) and *ShhGFPCRE/Smo*^*f/f*^ embryos. E11.5 and E12.5 explants were cultured for 6 and 9 days, respectively, in the presence of vehicle control (DMSO), retinoids [*all-trans* retinoic acid (RA) and the retinoic acid receptor γ (RARγ) and RARβ selective agonists, CD1530 and CD2341, respectively], the pan-RAR antagonist BMS493, or the retinoic acid synthesis inhibitor 4-diethylaminobenzaldehyde (DEAB) (S3 Table). The specimens were thereafter processed for paraffin embedding and immunostaining for K8 as described above. All sections of explants from the *ShhGFPCRE/Smo*^*f/f*^ mutants treated with DEAB or BMS493 were processed for K8 immunostaining. The E11.5 specimens cultured for 6 days were exposed to DMSO, BMS493 (10 μM or 12.5 μM), DEAB (20 μM during the first 3 days of culture and 10 μM during the last 3 days of culture), CD1530 (1 μM), CD2314 (1 μM) or RA (3 μM). The E12.5 explants cultured for 9 days were treated with DMSO or 10 μM BMS493.

Tongue and tail explants from E11.5 *RARE-hsp68-LacZ* embryos were cultured together in the same dishes for 6 or 2 days, respectively, in the presence of RA (3 μM; n = 2), CD1530 (1 μM, n = 1) and CD2314 (1 μM, n = 1). They were thereafter processed for *LacZ* staining following cryostat sectioning.

E12 explants were cultured for 2 days in the presence of retinoids, BMS493, or DEAB (S4 Table), and processed for *Shh in situ* hybridization. These were treated with DMSO, DEAB (25 μM), RA (3 μM), BMS493 (12.5 μM), CD1530 (0.5 μM, 1 μM or 1.5 μM) and CD2314 (0.5 μM, 1 μM or 1.5 μM). E12 explants from control embryos were cultured for 2 days in the presence of vehicle (DMSO; n = 4) or 0.2 μM SAG (n = 7), a Smoothened agonist [94], and processed for *Shh in situ* hybridization. The media containing freshly added compounds were changed daily.

RA, SAG, BMS493, and DEAB were from Sigma-Aldrich, St. Louis, MO, USA. CD1530 and CD2314 were from Tocris Bioscience, Abingdon, UK. All explants were cultured for the indicated periods at 37°C in an atmosphere of 5% CO_2_ in air. The culture medium consisted of DMEM/F-12 mix containing 2% fetal bovine serum, 2% vitamin A-free B27 supplement, 0.5% ITS, 0.5% glutamax, and penicillin/streptomycin (50 IU/ml and 50 μg/ml) from Life Technologies.

### RT-quantitative PCR

Tongues from *ShhCreER*^*T2*^*/Shh*^*f*^ mutants (first exposed to TAM at E10.5 or E11.5) and *ShhGFPCRE/Smo*^*f/f*^ mutants as well as their respective controls were dissected up to the level and including the circumvallate papilla. RNA was extracted using the RNAeasy Mini Kit (Qiagen), and cDNA was synthesized by reverse transcriptase (Qiagen). Quantitative PCR was performed using the iTAq Universal SYBR Green Supermix (Biorad) with the following primer sets: m*RARg* Forward 5'-GCAAGTACACCACGAACTCC, m*RARg* Reverse 3' AGGATGTCCAGACAAGCAGC; m*RARb* Forward 5'-GGAGAACTTGGGATCGGTGC m*RARb* Reverse 3'-TCTCGATGGCATTTTCCAGGC); m*Cyp26b1* Forward 5’- CCGTGAGAAGCTGCAGTGTA, m*Cyp26b1* Reverse 3’- GGGTTCCATCCTTCAGCTCC [95]; m*Ptch1* Forward 5’- CTAGCAATAGGGACCGCTCA m*Ptch1* Reverse 3’- GTCTCAGGGTAGCTCTCATAGC [96], and m*Actb* Forward 5'-AGAGGGAAATCGTGCGTGAC, m*Actb Reverse* 3'-CAATAGTGATGACCTGGCCGT). Student’s t-test was used for statistical analyses.

## Supporting information

S1 Text Tongues from control and mutant embryos studied. Description of genotypes and number of tongues from the various mutants and their respective controls assessed in this study.
(PDF)

S1 Fig Efficient ablation of *Smoothened* (*Smo*) in the lingual epithelium. (**A-D**) Frontal tongue section (A) and tongues/mandibles (B-D) from *ShhGFPCRE/R26R* embryos at E11.5 (B), E12.5 (A,C) and E13.5 (D) showing β-galactosidase activity, indicating the sites of CRE activity (blue). (**E-H’**) *Gli1* (E,F) and *Ptch1* (G,H) *in situ* hybridization (brown) in parasagittal tongue sections from E12.5 control (E,G) and *ShhGFPCRE/Smo*^*f/f*^ mutant (F,H) embryos. (E’-H’) Enlarged images of the boxed areas in (E-H). The dotted lines highlight the junction between the lingual epithelium and the lingual mesenchyme. The mutant lingual epithelium is devoid of *Gli1* and *Ptch1* expression, indicating efficient *Smo* ablation. (**I**) RT-qPCR analysis for *Ptch1* relative to *Actb* (β-actin) in tongues from E12.5 *ShhGFPCRE/Smo*^*f/f*^ mutants (n = 5) and controls (n = 5) showing downregulation of *Ptch1* levels (*P* = 0.0001; mean values ± SD) in the mutant tongues as compared to the control tongues. IME, intermolar eminence; T, tongue. Scale bars: 500 μm (B-D), 200 μm (E-H), 100 μm (A), and 50 μm (E’-H’).
(TIF)

S2 Fig *Wnt1-CRE/Smo*^*f/f*^ tongues lacking *Smo* in the lingual mesenchyme develop taste buds, and normal tongue development upon loss of SHH signaling after E12.5. **(A-D**) Sonic hedgehog (SHH; A,B) and Keratin 8 (K8; C,D) staining (dark purple) of frontal sections of E18.5 control (A,C) and *Wnt1-CRE/Smo*^*f/f*^ mutant (B,D) tongues showing normal differentiation of SHH-positive (+) and K8+ taste buds (TB) in the mutant tongues. The *Wnt1-CRE/Smo*^*f/f*^ tongues are severely reduced in size and cleft. (**E-J’**) Anti-K8-stained parasagittal tongue sections. Sections from E18.5 control (E) and *ShhCreER*^*T2*^*/Smo*^*f/f*^ (F) embryos first exposed to tamoxifen (TAM) at E12.5. Sections from E18.5 control (G) and *ShhCreER*^*T2*^*/Shh*^*f*^ (H) embryos first exposed to TAM at E12.5. Sections from control (I) and *K14-Cre/Smo*^*f/f*^ mutant (J) newborns (P0). (E’-J’) Enlarged images of the boxed areas in (E-J). The mutants show normal tongue development. (**K-N**) Tongues from E11.5 (K), E12.5 (L), E13.5 (M) and E15 (N) *K14-CRE/R26R* embryos after β-galactosidase histochemistry showing the sites of K14-CRE activity (blue). Robust K14-CRE activity in the lingual epithelium occurs after E12.5. T, tongue; TB, taste bud. Scale bars: 500 μm (E-N), 100 μm (A-D), and 50 μm (E’-J’).
(TIF)

S3 Fig *ShhGFPCRE/Smo*^*f/f*^ and tamoxifen-induced *ShhCreER*^*T2*^*/Shh*^*f*^ mutant tongues display enhanced *RARb* and *RARg* signals in the lingual epithelium. (**A-P**) Dark-field images showing *RARg* (A,B,E,F,I,J,M,N) and *RARb* (C,D,G,H,K,L,O,P) mRNA expression in frontal (A-D) and parasagittal (E-P) tongue sections. Hybridization signals appear as shiny dots. Sections from the same specimens were processed for *RARg* and *RARb* detection. (**A-L**) Sections from E12.5 (A-D), E13.5 (E-H) and E14.5 (I-L) control (A,C,E,G,I,K) and *ShhGFPCRE/Smo*^*f/f*^ mutant (B,D,F,H,J,L) embryos. Abnormal adhesion of the mutant tongue to the floor of the oral cavity (arrowheads in J and L). (**M-P**) Sections from E14.5 control (M,O) and *ShhCreER*^*T2*^*/Shh*^*f*^ mutant (N,P) embryos first exposed to tamoxifen at E10.5. All the mutants show enhanced *RARb* and *RARg* hybridization signals in the lingual epithelium. T, tongue. Scale bars: 500 μm.
(TIF)

S4 Fig *RARg*/RARγ are expressed in taste buds and lingual glands, and the *ShhGFPCRE/Smo*^*f/f*^ lingual epithelium exhibits enhanced RARγ immunoreactivity. (**A-N**) Parasagittal (A-J,L-N) and frontal (K) tongue sections after immunostaining for RAR**γ** protein (dark purple) and *in situ* hybridization for *RARg* mRNA (dark brown). (**A**,**B**) RAR**γ** staining in E11.5 control (A) and *ShhGFPCRE/Smo*^*f/f*^ mutant (B) tongues showing enhanced RAR**γ** staining in the lingual epithelium of the mutant. (**C-G**) RAR**γ** staining in E13.5 (C,D) and E14.5 (E-G) control (C,E) and *ShhGFPCRE/Smo*^*f/f*^ mutant (D,F,G) tongues. Enhanced RAR**γ** staining in the lingual epithelium of the mutants, except in epithelial foci (arrow in G). Taste buds (TB) in developing fungiform papillae are RAR**γ**-positive (+) (E). (**H,I**) Anti-RAR**γ**-stained sections of E16.5 control (H) and *ShhGFPCRE/Smo*^*f/f*^ mutant (I) tongues showing strong RAR**γ** staining in the lingual epithelium and in TBs within fungiform papillae (FuP; H,I). The mutant tongue displays epithelial foci with weak RARγ staining (arrows in I). (**J,K**) RARγ protein (J) and *RARg* mRNA (K) expression in TBs of fungiform papillae in a control newborn (P0). (**L**) RARγ+ developing von Ebner’s glands (vEG) in a control tongue at E16.5. (**M,N**) RARγ+ posterior lingual glands (pLGs) and RARγ+ heterotopic glands (HG) in control (M) and *ShhGFPCRE/Smo*^*f/f*^ mutant (N) tongues at P0. T, tongue. Scale bars: 200 μm (C,D), 100 μm (A,B,E,F,H,I,J,M,N), and 50 μm (G,K,L).
(TIF)

S5 Fig RALDH1-3 proteins are expressed in the developing tongue. (**A-Z**) Parasagittal tongue (T) sections from controls and *ShhGFPCRE/Smo*^*f/f*^ mutants after immunostaining (dark purple) for RALDH1, RALDH2 and RALDH3. (**A-F**) RALDH1-3 distribution in E11.5 control (A,C,E) and mutant (B,D,F) tongues. The lingual epithelium (LE) of the posterior tongue (arrows in A and B) is RALDH1-positive (+). The posterior lingual mensenchyme is RALDH2+ (arrows in C and D). Enhanced RALDH3 staining in the LE of the mutant (arrows in F) relative to that of the control (arrows in E). (**G-L**) RALDH1-3 distribution in E12.5 control (G,I,K) and mutant (H,J,L) tongues. The lingual periderm, the developing circumvallate papilla (CvP), and the epithelium of the pharyngeal tongue are RALDH1+ (G,H). The LE and subsets of mesenchymal cells are RALDH2+ (I,J). The LE of the tip of the tongue and muscle fibers are RALDH3+ (K,L). (**M**-**R**) RALDH1-3 distribution in E13.5 control (M,O,Q) and mutant (N,P,R) tongues. The periderm (M,N) and a nascent heterotopic gland (nHG; N) are RALDH1+. Mesenchymal cells located between muscle fibers, and the LE are RALDH2+ (O,P). Fungiform placodes (arrows in Q and R) and muscle fibers are RALDH3+ (Q,R). (**S-X**) RALDH1-3 distribution in E16.5 control (S,U,W) and mutant (T,V,X) tongues. Suprabasal epithelial cells and taste buds (TB) of fungiform papillae are RALDH1+ (S,T). Enhanced RALDH2 staining in developing heterotopic glands in the mutant (arrows in V). Cells wrapping nerve fibers innervating fungiform papillae (arrows in W and X), and suprabasal epithelial cells are RALDH3+ (W,X). (**Y,Z**) RALDH1 staining in tongues from newborn (P0; Y) and adult (Z) mice showing RALDH1+ TBs in fungiform papillae. BA2, branchial arch 2; PT, pharyngeal tongue. Scale bars: 200 μm (A-P), 100 μm (Q,R,U-X,Z), and 50 μm (S,T,Y).
(TIF)

S6 Fig The orthotopic and heterotopic lingual glands express RALDH1-3 proteins. (**A-L**) Parasagittal tongue sections from control and *ShhGFPCRE/Smo*^*f/f*^ mutant newborns (P0) after immunohistochemistry (dark purple) for RALDH1 (A-D), RALDH2 (E-H) and RALDH3 (I-L). Sections across the pharyngeal tongue of controls (A,E,I) showing RALDH1-3 expression in the orthotopic posterior lingual glands (pLG). Sections across control (B,F,J) and mutant (C,G,K) tongues at the level of the circumvallate papilla showing RALDH1-3 protein expression in von Ebner’s glands (vEG). vEGs in the mutants are relatively overgrown and exhibit mucous metaplasia (mucous cells are filled with a light blue material). Sections across the oral tongue of mutants (D,H,L) showing RALDH1-3 protein expression in the heterotopic lingual glands (HG). Scale bars: 100 μm (A-L).
(TIF)

S7 Fig Retinoids induce glandular metaplasia, and inhibitors of retinoic acid signaling inhibit lingual gland formation *in vitro*. (**A-K**) Anti-Keratin 8 (K8)–stained (dark purple) frontal sections across the oral tongue (T) in E11.5 mandibular arch explants cultured *in vitro* for 6 days. (**A-E**) Explants from control embryos (without the *CRE* and/or the *Smo* floxed alleles) treated with vehicle (DMSO; A) and 3 μM *all trans*-retinoic acid (RA; B). Explants from control embryos treated with DMSO (C), 1 μM CD1530 (D) and 1 μM CD2314 (E). The retinoids induced glandular metaplasia, *i*.*e*. formation of heterotopic glands (HG) in the oral tongue, and promoted salivary gland (SG) formation, but they inhibited development of Meckel’s cartilage (M) and other mandibular structures. (**F-K**) Explants from E11.5 *ShhGFPCRE/Smo*^*f/f*^ mutants treated with DMSO (F,I), DEAB (G,H) and 12.5 μM BMS493 (J,K). DEAB was used at 20 μM and 10 μM during the first and last 3 days of culture, respectively. (H) and (K) are sections across the anterior-most part of the oral tongue of the specimens shown in (G) and (J), respectively. The DMSO-treated mutant tongues recapitulated the *in vivo* defects, including formation of K8-positive (+) heterotopic glands (HG), K8+ ectopic Merkel cells (arrowheads in F and I), and K8-negative (–) squamous epithelial foci (ef). DEAB and BMS493 inhibited heterotopic gland formation (G,H,J,K), but failed to inhibit Merkel cell metaplasia (arrowheads in H and J point at the ectopic Merkel cells) and formation of K8(–) epithelial foci. (**L-M’**) Anti-K8-stained oblique sections across the circumvallate papilla (CvP) and posterior lingual glands (pLG) of E12.5 mandible/tongue explants from control embryos after 9 days of *in vitro* culture in the presence of vehicle (DMSO; L) and 10 μM BMS493 (M). (L’) and (M’) are enlarged images of the boxed areas in (L) and (M), respectively. BMS493 inhibited development of posterior lingual glands (arrow in M) and von Ebner’s glands (vEG). Scale bars: 200 μm (A-K, L,M) and 50 μm (L’,M’).
(TIF)

S8 Fig Absence of *RARE-hsp68-LacZ* activity in the lingual epithelium *in vivo* and after *in vitro* treatment with retinoids. (**A-I**) β-galactosidase (β-gal) histochemistry for visualization of *RARE-hsp68-LacZ* transgene activity (blue) in cryostat sections. (**A-F**) Parasagittal tongue (T) sections from control (*ShhGFPCRE/RARE-LacZ*) and mutant (*ShhGFPCRE/Smo*^*f/f*^*/RARE-LacZ*) embryos at E11.5 (A,B), E12.5 (C,D) and E17.5 (E,F). The insets in (E) and (F) are enlarged images of the boxed areas in (E) and (F), respectively, and show β-gal-positive (+) desquamating suprabasal epithelial cells. Weak β-gal activity in subsets of epithelial and mesenchymal cells at the junction between branchial arches 1& 2 (arrows in A and B). The quasi-totality of the lingual epithelium in the controls and mutants is β-gal-negative (A-F). The heterotopic glands (HG) in the mutant are β-gal-negative (F). (**G-I**) Sections across the oral tongue/mandible (G,I) and tail (H) from E11.5 *ShhGFPCRE/RARE-LacZ* embryos after *in vitro* culture for 6 (G,I) or 2 (H) days in the presence of 3 μM *all-trans* retinoic acid (RA; G,H) or 1 μM CD1530 (I). The tail explant was cultured in the same dish as the explant shown in (G). In contrast to the tail, neither the salivary glands (SG) nor the retinoid-induced heterotopic glands (HG) in the oral tongue are β-gal+. Scale bars: 500 μm (E,F) and 200 μm (A-D,G-I).
(TIF)

S9 Fig Patterning and cell fate specification in the lingual epithelium, and tongue growth and morphogenesis require SHH inputs before E12.5. (**A**) Before E12.5, SHH signaling controls antero-posterior patterning and cell fate specification in the LE of the oral tongue, whereby SHH inhibits the development of minor salivary glands and taste placodes (thick red arrow). SHH fulfills this function by abating retinoic acid (RA) signaling (thin red arrow) in the interplacodal epithelium through maintenance and/or reinforcement of expression of transcripts encoding the RA catabolic enzymes CYP26A1 and CYP26C1 (thin green arrow). SHH input in the LE is also required to inhibit Merkel cell specification (thick red arrow) through a yet to be determined mechanism (blue arrow). SHH is known to antagonize canonical Wnt signaling (thin red arrow), a promoter of taste placode induction. This is achieved, at least in part, through inhibition of *Wnt10b* expression. (**B**) Loss of SHH signaling in the LE before E12.5 causes loss of expression of *Cyp26a1* and *Cyp26c1* expression (thin red arrow). Hence, unabated RA signaling (thin green arrow), triggered by the highly diffusible small molecule RA (not depicted), causes overproduction of oversized taste buds, sustained induction of taste placodes, and development of sero-mucous minor salivary glands (thick green arrow). Furthermore, absence of epithelial SHH signaling prompts the LE to aberrantly generate Merkel cells (thick green arrow), the underlying mechanism of which is unknown (blue arrow). Loss of epithelial SHH inputs also leads to anachronistic and expanded expression of *Wnt10b* (thin green arrow). (**C**) Before E12.5, SHH signaling in the lingual mesenchyme (LM) is crucial (green arrow) for growth and morphogenesis of the tongue but is not required for cell fate specification in the LE. (**D**) Loss of SHH signaling in the LM before E12.5 causes altered growth (microglossia) and morphogenesis (clefting) of the tongue (thick red arrow).
(TIF)

S1 Table Quantification of taste buds and ectopic Merkel cells in control and *ShhGFPCRE/Smo*^*f/f*^ tongues after immunofluorescence. (PDF)

S2 Table Quantification of taste buds innervated by gustatory neurites in controls and *ShhCreER*^*T2*^ */Shh*^*f*^ mutants. (PDF)

S3 Table Retinoids induce glandular metaplasia and retinoic acid signaling inhibitors abrogate heterotopic and orthotopic lingual gland formation. (PDF)

S4 Table Retinoids promote and inhibitors of retinoic acid signaling inhibit fungiform placode formation after a 2-day treatment of tongue explants. (PDF)
