# Supplementary material for: Cell fate specification in the lingual epithelium is controlled by antagonistic activities of Sonic hedgehog and retinoic acid
Source: PLoS Genet. 2017 Jul 17;13(7):e1006914. doi: 10.1371/journal.pgen.1006914 (PMC5536368; doi:10.1371/journal.pgen.1006914)
Supplement: S1 Text — Description of genotypes and number of tongues from the various mutants and their respective controls assessed in this study. (PDF) [file pgen.1006914.s001.pdf]

S1 Text. Tongues from control and mutant embryos studied

Description of genotypes and number of tongues from the various mutants and their respective controls assessed in this study.

ShhGFPCRE/Smo$^{f/f}$ mutants

Alcian blue van Gieson: n=5 at E17.5 to birth (P0).

Immunohistochemistry: Homer1 (n=1 at P0), Keratin 6 (n=2 at P0) Keratin 15 (n=1 at P0), Keratin 8 (n=17 at E17.5 to P0), Rab3c (n=2 at P0), RALDH1 (n=11 at E11.5 to P0), RALDH2 (n=9 at E11.5 to P0), RALDH3 (n=8 at E11.5 to P0), RARγ (n=11 at E11.5 to P0), SHH (n=11 at E12.5 to P0), and SOX2 (n=4 at P0).

In situ hybridization (sections): *Cyp26a1* (n=2 at E12.5 to E13.5), *Cyp26c1* (n=3 at E12.5 to E14), *Gli1* [(n=1 at E12.5 (oligonucleotide probes); n=2 at E12.5 to E14.5 (riboprobes)], *Ptch1* [n=1 (oligonucleotide probes); n=2 (riboprobe) at E12.5], *RARb* (n=4 at E12.5 to E14.5), *RARg* [(n=3 at E12.5 to E13.5 (oligonuclotide probes); n=4 at E12.5 to E14.5 (riboprobe)], *Shh* (n=3 at E14.5 to E18.5), and *Wnt10b* (n=3 at E17.5 to P0).

Whole-mount in situ hybridization: *Cyp26a1* (n=5 at E11.5 to E14), *Cyp26c1* (n=2 at E11.5 to E12.5-E13), and *Shh* (n=6 at E14.5 to E15.5).

GFP imaging: n=2 at E14.5.

Controls for *ShhGFPCRE/Smo*$^{f/f}$ mutants

Alcian blue van Gieson: n=4 at E17.5 to P0.

Immunohistochemistry: Homer1 (n=1 at P0 and n=1 adult), Keratin 6 (n=3 at P0) Keratin 15 (n=1 at P0), Keratin 8 (n=21 at E17.5 to P0), Rab3c (n=6 at P0), RALDH1 (n=11 at E11.5 to P0, n=1 at P15, n=1 adult), RALDH2 (n=10 at E11.5 to P0, n=1 at P15, n=1 adult), RALDH3 (n=13 at E11.5 to P0, n=1 at P15 n=1 adult), RARγ (n=12 at E12.5 to P0), SHH (n=9 at E12.5 to P0), and SOX2 (n=6 at P0),.

In situ hybridization (sections): *Cyp26a1* (n=3 at E12.5 to E13.5), *Cyp26c1* (n=3 at E12.5 to E14), *Gli1* [(n=1 at E12.5 (oligonucleotide probes); n=2 at E12.5 to E14.5 (riboprobes)], *Ptch1* [n=1 (oligonucleotide probes); n=2 (riboprobe) at E12.5], *RARb* (n=4 at E12.5 to E14.5), *RARg* [(n=3 at E12.5 to E13.5 and n=1 at P0 (oligonucleotide

probes); n=4 at E12.5 to E14.5 (riboprobe)], *Shh* (n=3 at E14.5 to E18.5), and *Wnt10b* (n=3 at E17.5 to P0).

Whole-mount in situ hybridization: *Cyp26a1* (n=11 at E11 to E14.5), *Cyp26c1* (n=6 at E11.5 to E12.5-E13), and *Shh* (n=14 at E14.5 to E15.5).

GFP imaging: n=2 at E14.5.

E10.5 Tamoxifen-induced *ShhCreERT2/Shh^f* mutants

Immunohistochemistry: Keratin 8 (n=3 at E15.5 to E17.5), Rab3c (n=1 at E17.5), SHH (n=2 at E15.5), and SOX2 (n=1 at E15).

In situ hybridization (sections): *Cyp26a1* (n=1 at E13.5), *Cyp26c1* (n=1 at E14), *Gli1* (n=2 at E13.5), *RARb* (n=1 at E14.5), *RARg* [n=1 at E13.5 (oligonucleotide probes); n=1 at E14.5 (riboprobe)], and *Wnt10b* (n=2 at E13.5 to E15.5).

Whole-mount in situ hybridization: *Cyp26a1* (n=2 at E14.5), *Cyp26c1* (n=2 at E14-E14.5), and *Shh* (n=3 at E15).

E10.5 Tamoxifen-induced controls for *ShhCreERT2/Shh^f* mutants

Immunohistochemistry: Keratin 8 (n=3 at E15.5 to E17.5), Rab3c (n=1 at E17.5), SHH (n=1 at E15.5), and SOX2 (n=1 at E15).

In situ hybridization (sections): *Cyp26a1* (n=1 at E13.5), *Cyp26c1* (n=1 at E14), *Gli1* (n=2 at E13.5), *RARb* (n=1 at E14.5), *RARg* [(n=1 at E13.5 (oligonucleotide probes); n=1 at E14.5 (riboprobe)], and *Wnt10b* (n=2 at E13.5 to E15.5).

Whole-mount in situ hybridization: *Cyp26a1* (n=2 at E14.5), *Cyp26c1* (n=2 at E14-E14.5), and *Shh* (n=6 at E15).

E11.5 Tamoxifen-induced *ShhCreERT2/Shh^f* mutants

Immunohistochemistry: Homer1 (n=1 at E18), Keratin 8 (n=6 at E18.5), Rab3c (n=1 at E18), RARγ (n=1 at E16.5), SHH (n=3 at E16), and SOX2 (n=1 at E16).

In situ hybridization (sections): *Cyp26a1* (n=1 at E14), *Gli1* (n=1 at E14.5), *RARg* (n=2 at E13.5 to E14), and *Wnt10b* (n=1 at E17.5).

Whole-mount in situ hybridization: *Cyp26a1* (n=2 at E15), *Cyp26c1* (n=2 at E15), *Shh* (n=4 at E15.5), and *Shh-exon2* (n=2 at E13.5 to E15).

E11.5 Tamoxifen-induced controls for *ShhCreERT2/Shh^{f}* mutants

Immunohistochemistry: Homer1 (n=1 at E18), Keratin 8 (n=4 at E18.5), Rab3c (n=1 at E18), RARγ (n=1 at E16.5), SHH (n=3 at E16), and SOX2 (n=1 at E16).

In situ hybridization (sections): *Cyp26a1* (n=1 at E14), *Gli1* (n=1 at E14.5), *RARg* (n=2 at E13.5 to E14), and *Wnt10b* (n=1 at E17.5).

Whole-mount in situ hybridization: *Cyp26a1* (n=2 at E15), *Cyp26c1* (n=2 at E15), *Shh* (n=3 at E15.5), and *Shh*-exon2 (n=2 at E13.5 to E15).

Controls and *Wnt1-CRE/Smo$^{f/f}$* mutants

Alcian Blue van Gieson: n=2 controls and n=2 mutants at E18.5.

Immunohistochemistry: SHH and Keratin 8 (n=2 controls and n=2 mutants at E18.5).

E12.5-tamoxifen-induced Controls and *ShhCreERT2/Shh^{f} mutants*

Immunohistochemistry: Keratin 8 (n=3 controls and n=3 mutants at E18.5).

Whole-mount in situ hybridization: *Cyp26a1* (n=3 at E14.5 for each genotype), and *Cyp26c1* (n=3 at E14.5 for each genotype).

E12.5 tamoxifen-induced controls and *ShhCreERT2/Smo$^{f/f}$* mutants

Immunohistochemistry: Keratin 8 (n=5 controls and n=5 mutants at E18.5).

Control and *K14-Cre/Smo$^{f/f}$* mutants

Immunohistochemistry: Keratin 8 (n=4 control and n=4 mutant newborns).

***ShhGFPCRE/RARE-hsp68-LacZ* and *ShhGFPCRE/Smo$^{f/f}$/RARE-hsp68-LacZ* embryos**

β-galactosidase histochemistry: n=3 at E11.5 to E17.5 for each genotype.

***ShhGFPCRE/R26R* embryos**

β-galactosidase histochemistry: n=5 at E11.5 to E13.5.

***K14-CRE/R26R* embryos**

β-galactosidase histochemistry: n=14 at E11.5 to E15.
