# Supplementary figures and images for: Cell fate specification in the lingual epithelium is controlled by antagonistic activities of Sonic hedgehog and retinoic acid
Source: PLoS Genet. 2017 Jul 17;13(7):e1006914. doi: 10.1371/journal.pgen.1006914 (PMC5536368; doi:10.1371/journal.pgen.1006914)

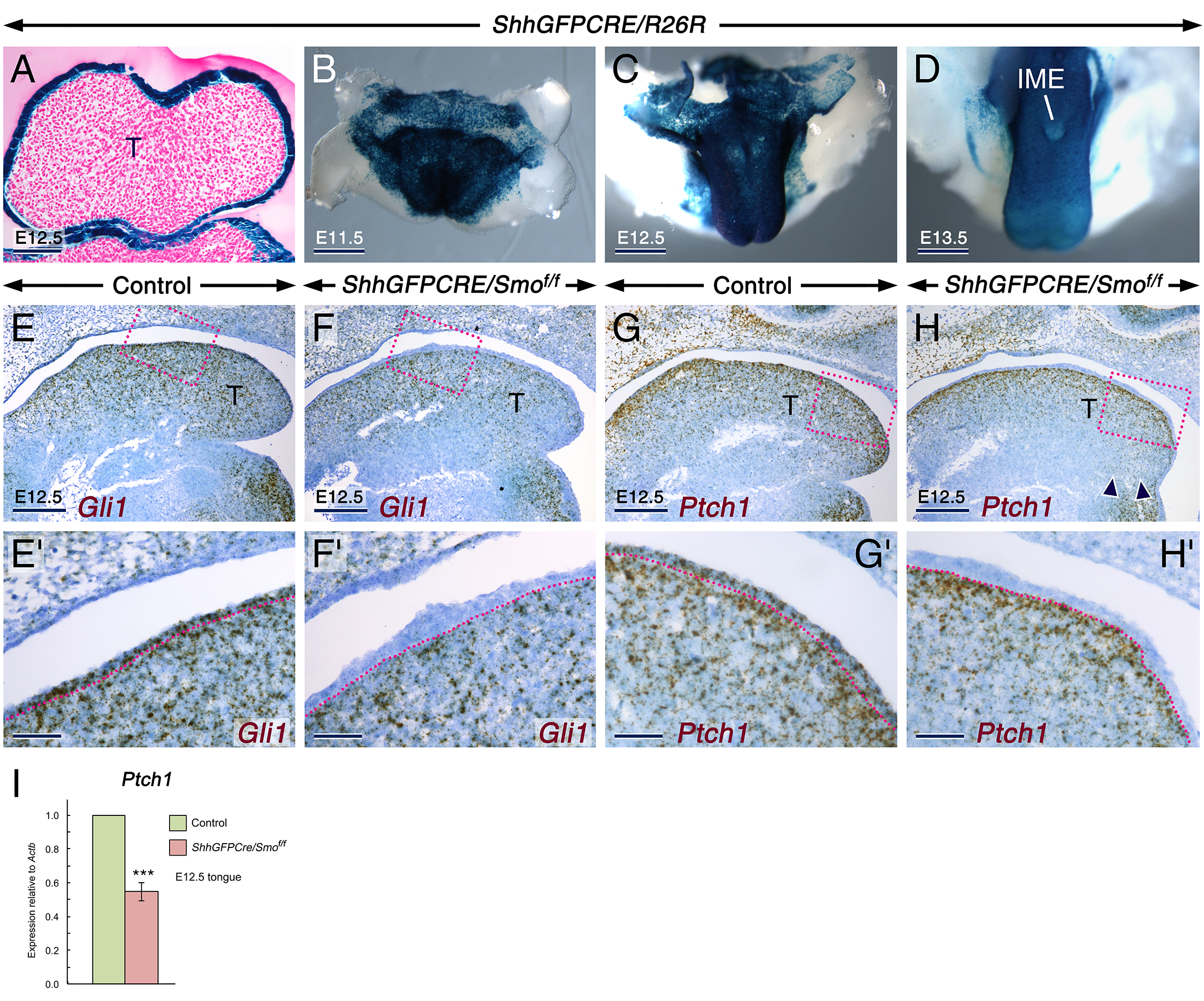

Supplement: S1 Fig — (A-D) Frontal tongue section (A) and tongues/mandibles (B-D) from ShhGFPCRE/R26R embryos at E11.5 (B), E12.5 (A,C) and E13.5 (D) showing β-galactosidase activity, indicating the sites of CRE activity (blue). (E-H’) Gli1 (E,F) and Ptch1 (G,H) in situ hybridization (brown) in parasagittal tongue sections from E12.5 control (E,G) and ShhGFPCRE/Smof/f mutant (F,H) embryos. (E’-H’) Enlarged images of the boxed areas in (E-H). The dotted lines highlight the junction between the lingual epithelium and the lingual mesenchyme. The mutant lingual epithelium is devoid of Gli1 and Ptch1 expression, indicating efficient Smo ablation. (I) RT-qPCR analysis for Ptch1 relative to Actb (β-actin) in tongues from E12.5 ShhGFPCRE/Smof/f mutants (n = 5) and controls (n = 5) showing downregulation of Ptch1 levels (P = 0.0001; mean values ± SD) in the mutant tongues as compared to the control tongues. IME, intermolar eminence; T, tongue. Scale bars: 500 μm (B-D), 200 μm (E-H), 100 μm (A), and 50 μm (E’-H’). (TIF) [file pgen.1006914.s002.tif]

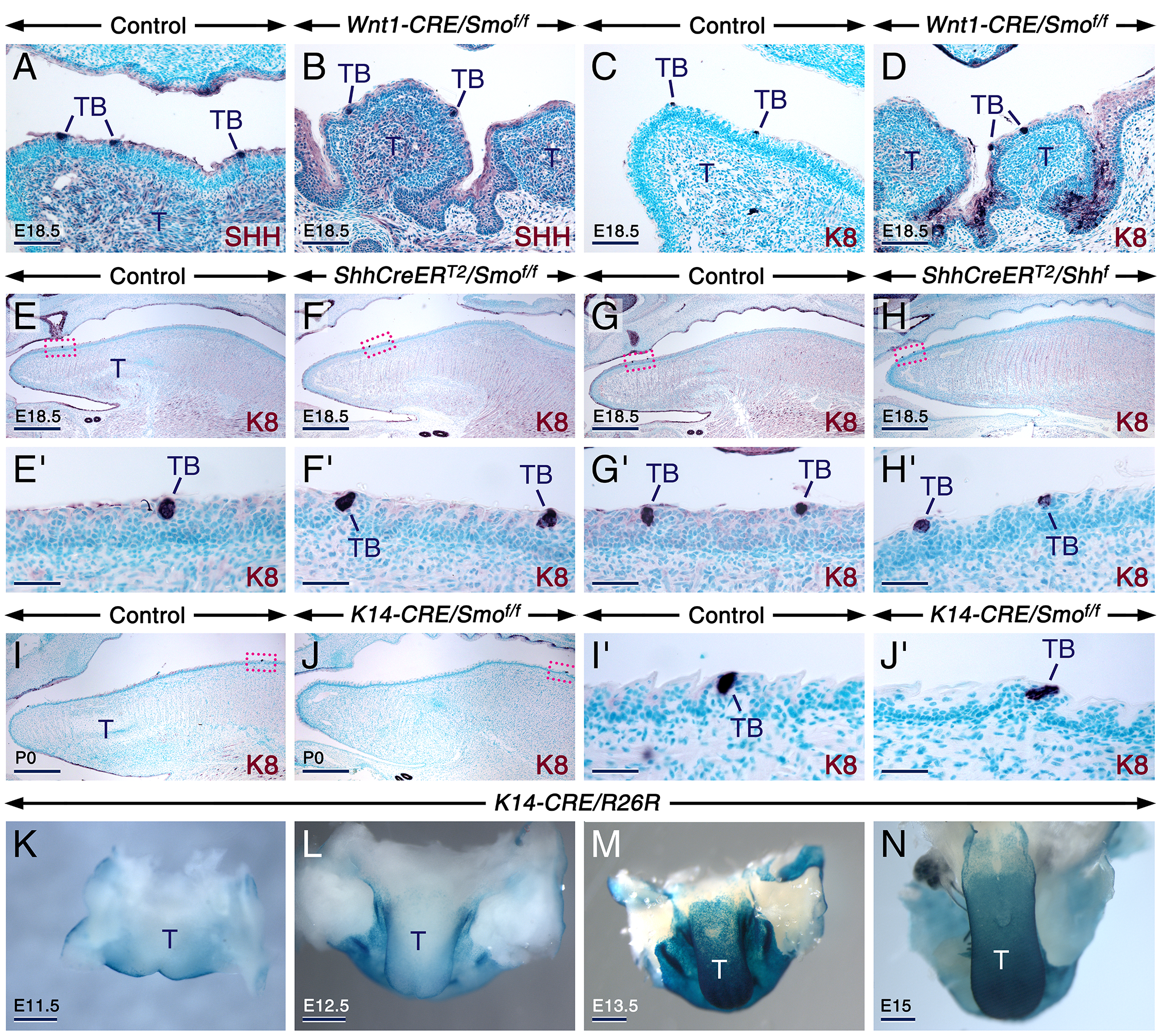

Supplement: S2 Fig — (A-D) Sonic hedgehog (SHH; A,B) and Keratin 8 (K8; C,D) staining (dark purple) of frontal sections of E18.5 control (A,C) and Wnt1-CRE/Smof/f mutant (B,D) tongues showing normal differentiation of SHH-positive (+) and K8+ taste buds (TB) in the mutant tongues. The Wnt1-CRE/Smof/f tongues are severely reduced in size and cleft. (E-J’) Anti-K8-stained parasagittal tongue sections. Sections from E18.5 control (E) and ShhCreERT2/Smof/f (F) embryos first exposed to tamoxifen (TAM) at E12.5. Sections from E18.5 control (G) and ShhCreERT2/Shhf (H) embryos first exposed to TAM at E12.5. Sections from control (I) and K14-Cre/Smof/f mutant (J) newborns (P0). (E’-J’) Enlarged images of the boxed areas in (E-J). The mutants show normal tongue development. (K-N) Tongues from E11.5 (K), E12.5 (L), E13.5 (M) and E15 (N) K14-CRE/R26R embryos after β-galactosidase histochemistry showing the sites of K14-CRE activity (blue). Robust K14-CRE activity in the lingual epithelium occurs after E12.5. T, tongue; TB, taste bud. Scale bars: 500 μm (E-N), 100 μm (A-D), and 50 μm (E’-J’). (TIF) [file pgen.1006914.s003.tif]

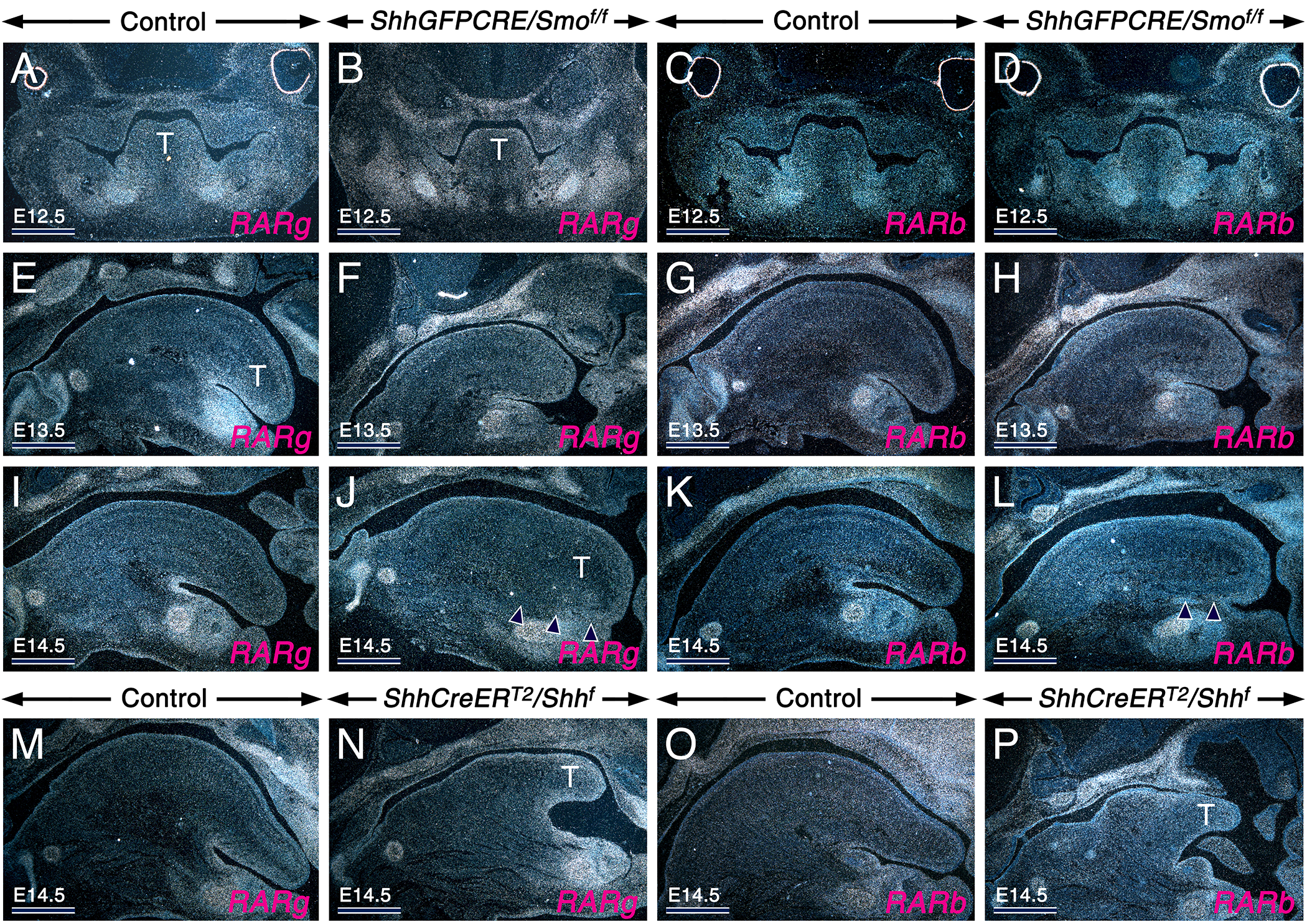

Supplement: S3 Fig — (A-P) Dark-field images showing RARg (A,B,E,F,I,J,M,N) and RARb (C,D,G,H,K,L,O,P) mRNA expression in frontal (A-D) and parasagittal (E-P) tongue sections. Hybridization signals appear as shiny dots. Sections from the same specimens were processed for RARg and RARb detection. (A-L) Sections from E12.5 (A-D), E13.5 (E-H) and E14.5 (I-L) control (A,C,E,G,I,K) and ShhGFPCRE/Smof/f mutant (B,D,F,H,J,L) embryos. Abnormal adhesion of the mutant tongue to the floor of the oral cavity (arrowheads in J and L). (M-P) Sections from E14.5 control (M,O) and ShhCreERT2/Shhf mutant (N,P) embryos first exposed to tamoxifen at E10.5. All the mutants show enhanced RARb and RARg hybridization signals in the lingual epithelium. T, tongue. Scale bars: 500 μm. (TIF) [file pgen.1006914.s004.tif]

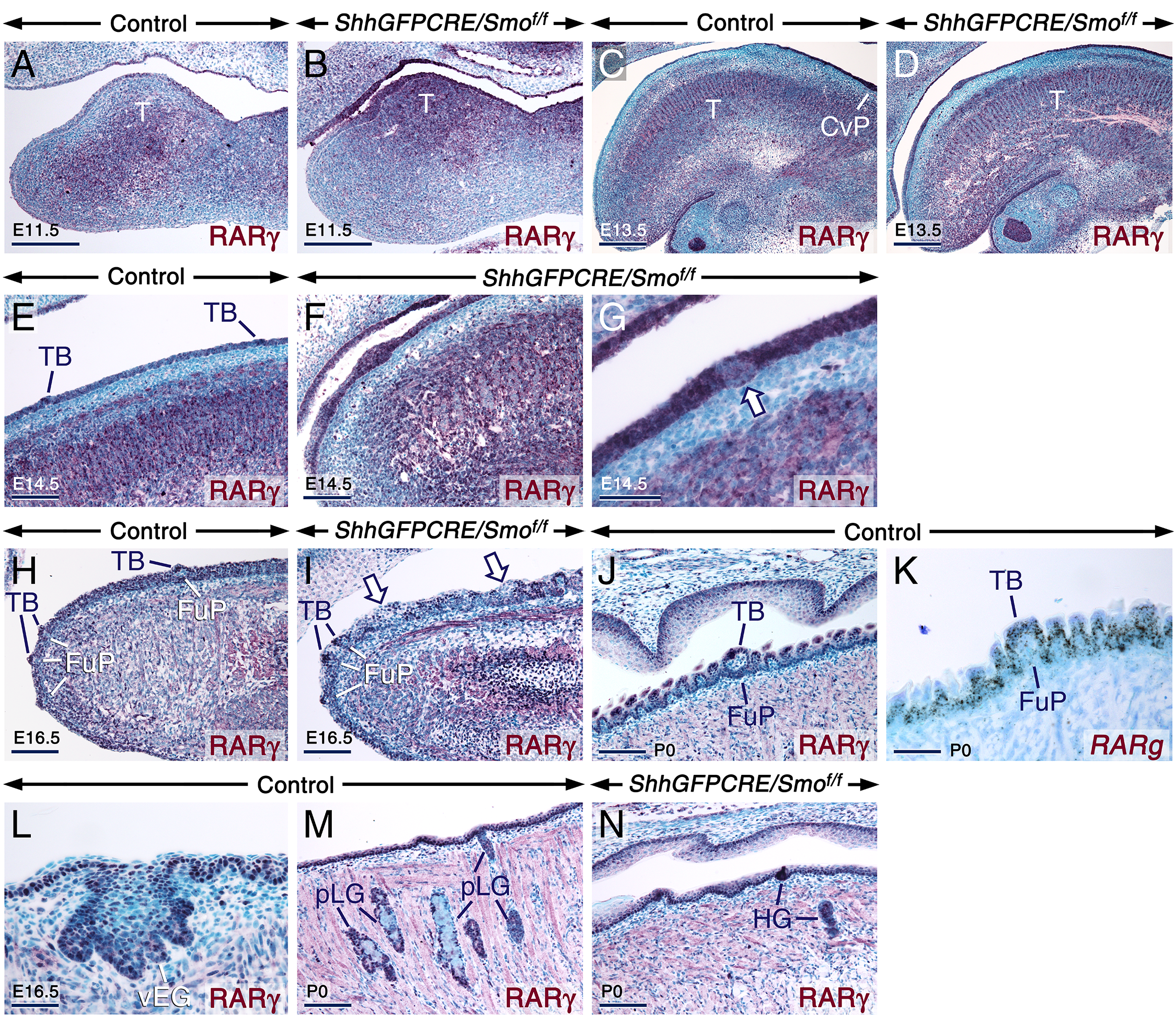

Supplement: S4 Fig — (A-N) Parasagittal (A-J,L-N) and frontal (K) tongue sections after immunostaining for RARγ protein (dark purple) and in situ hybridization for RARg mRNA (dark brown). (A,B) RARγ staining in E11.5 control (A) and ShhGFPCRE/Smof/f mutant (B) tongues showing enhanced RARγ staining in the lingual epithelium of the mutant. (C-G) RARγ staining in E13.5 (C,D) and E14.5 (E-G) control (C,E) and ShhGFPCRE/Smof/f mutant (D,F,G) tongues. Enhanced RARγ staining in the lingual epithelium of the mutants, except in epithelial foci (arrow in G). Taste buds (TB) in developing fungiform papillae are RARγ-positive (+) (E). (H,I) Anti-RARγ-stained sections of E16.5 control (H) and ShhGFPCRE/Smof/f mutant (I) tongues showing strong RARγ staining in the lingual epithelium and in TBs within fungiform papillae (FuP; H,I). The mutant tongue displays epithelial foci with weak RARγ staining (arrows in I). (J,K) RARγ protein (J) and RARg mRNA (K) expression in TBs of fungiform papillae in a control newborn (P0). (L) RARγ+ developing von Ebner’s glands (vEG) in a control tongue at E16.5. (M,N) RARγ+ posterior lingual glands (pLGs) and RARγ+ heterotopic glands (HG) in control (M) and ShhGFPCRE/Smof/f mutant (N) tongues at P0. T, tongue. Scale bars: 200 μm (C,D), 100 μm (A,B,E,F,H,I,J,M,N), and 50 μm (G,K,L). (TIF) [file pgen.1006914.s005.tif]

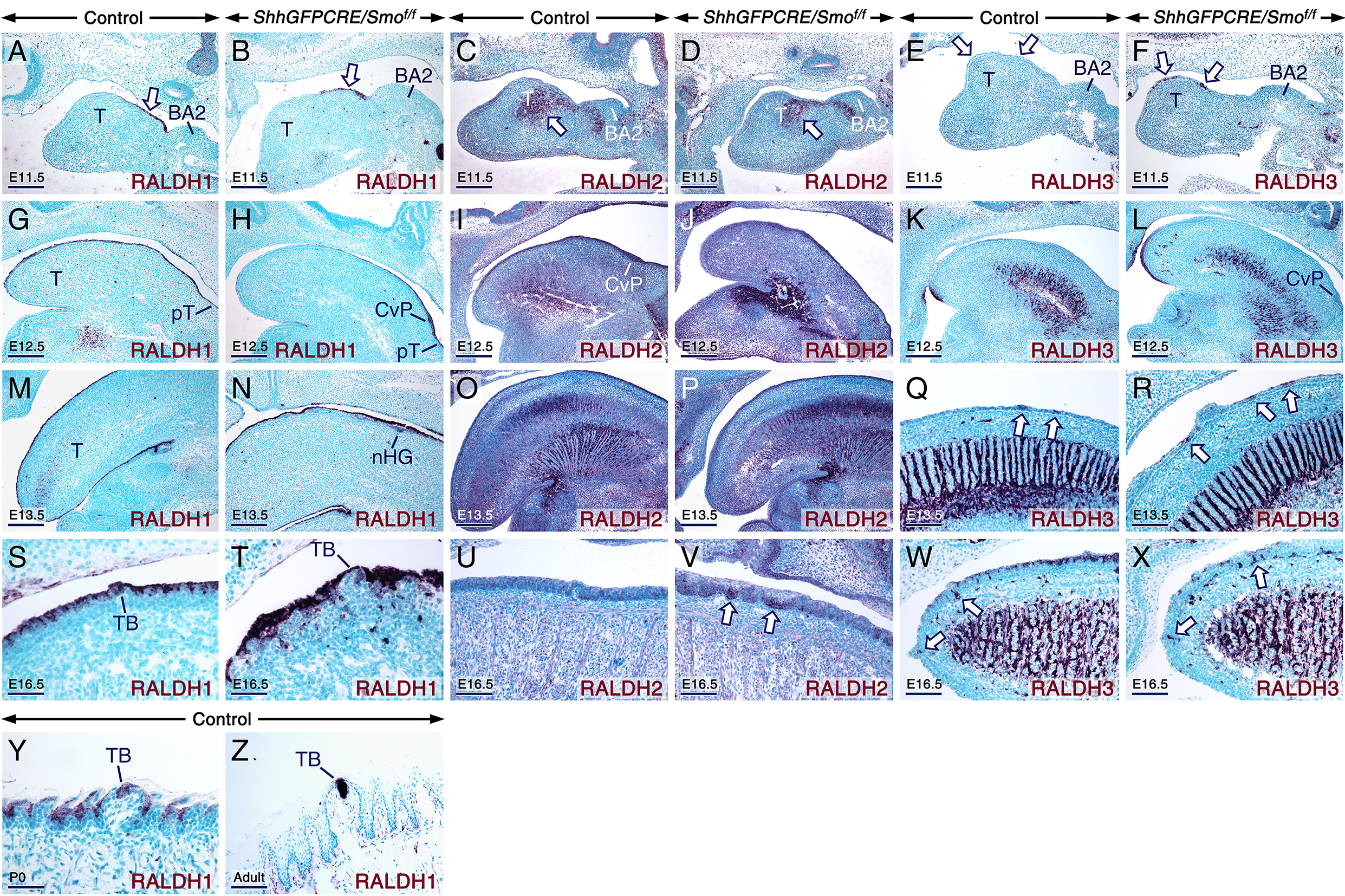

Supplement: S5 Fig — (A-Z) Parasagittal tongue (T) sections from controls and ShhGFPCRE/Smof/f mutants after immunostaining (dark purple) for RALDH1, RALDH2 and RALDH3. (A-F) RALDH1-3 distribution in E11.5 control (A,C,E) and mutant (B,D,F) tongues. The lingual epithelium (LE) of the posterior tongue (arrows in A and B) is RALDH1-positive (+). The posterior lingual mensenchyme is RALDH2+ (arrows in C and D). Enhanced RALDH3 staining in the LE of the mutant (arrows in F) relative to that of the control (arrows in E). (G-L) RALDH1-3 distribution in E12.5 control (G,I,K) and mutant (H,J,L) tongues. The lingual periderm, the developing circumvallate papilla (CvP), and the epithelium of the pharyngeal tongue are RALDH1+ (G,H). The LE and subsets of mesenchymal cells are RALDH2+ (I,J). The LE of the tip of the tongue and muscle fibers are RALDH3+ (K,L). (M-R) RALDH1-3 distribution in E13.5 control (M,O,Q) and mutant (N,P,R) tongues. The periderm (M,N) and a nascent heterotopic gland (nHG; N) are RALDH1+. Mesenchymal cells located between muscle fibers, and the LE are RALDH2+ (O,P). Fungiform placodes (arrows in Q and R) and muscle fibers are RALDH3+ (Q,R). (S-X) RALDH1-3 distribution in E16.5 control (S,U,W) and mutant (T,V,X) tongues. Suprabasal epithelial cells and taste buds (TB) of fungiform papillae are RALDH1+ (S,T). Enhanced RALDH2 staining in developing heterotopic glands in the mutant (arrows in V). Cells wrapping nerve fibers innervating fungiform papillae (arrows in W and X), and suprabasal epithelial cells are RALDH3+ (W,X). (Y,Z) RALDH1 staining in tongues from newborn (P0; Y) and adult (Z) mice showing RALDH1+ TBs in fungiform papillae. BA2, branchial arch 2; PT, pharyngeal tongue. Scale bars: 200 μm (A-P), 100 μm (Q,R,U-X,Z), and 50 μm (S,T,Y). (TIF) [file pgen.1006914.s006.tif]

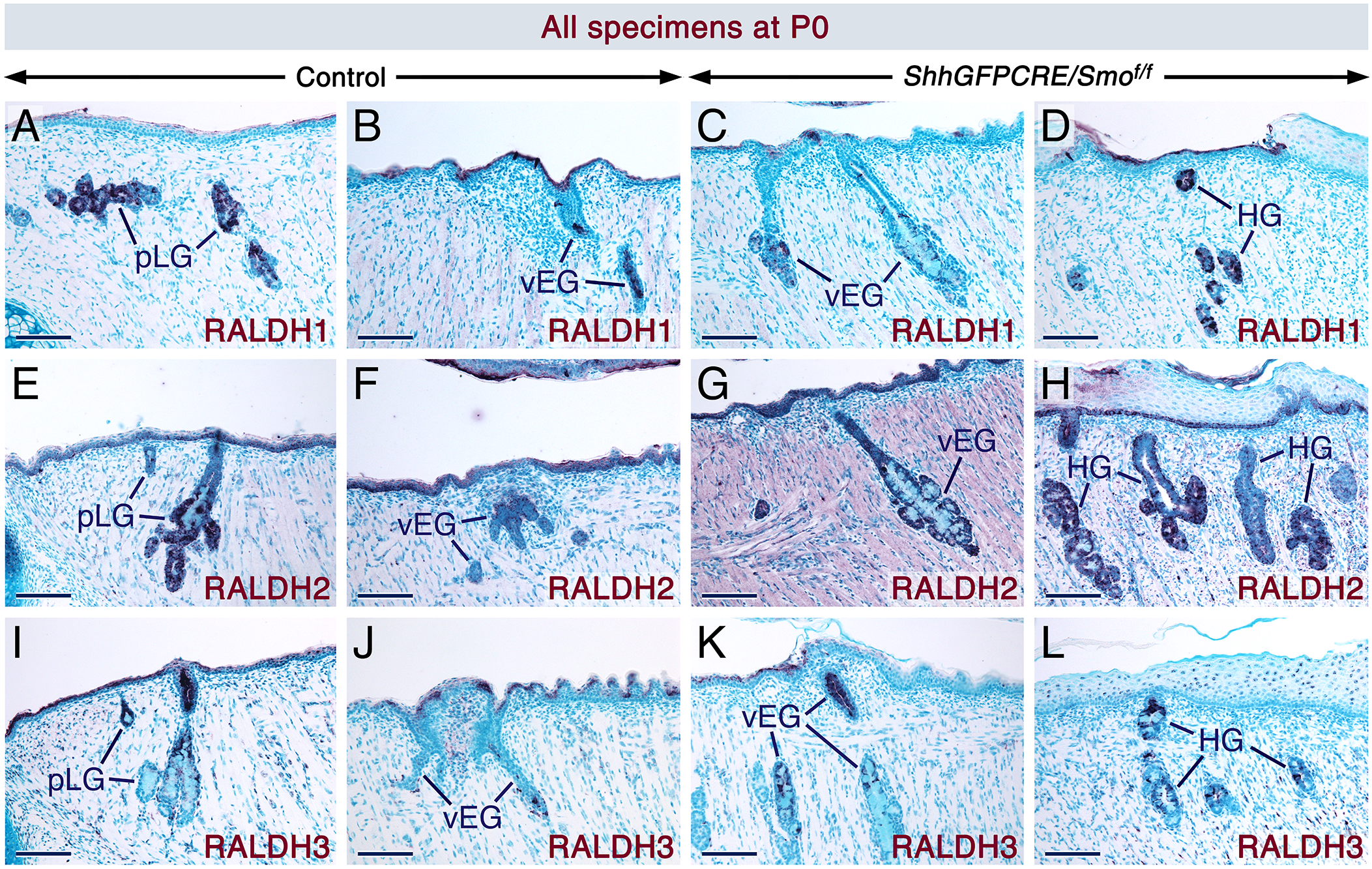

Supplement: S6 Fig — (A-L) Parasagittal tongue sections from control and ShhGFPCRE/Smof/f mutant newborns (P0) after immunohistochemistry (dark purple) for RALDH1 (A-D), RALDH2 (E-H) and RALDH3 (I-L). Sections across the pharyngeal tongue of controls (A,E,I) showing RALDH1-3 expression in the orthotopic posterior lingual glands (pLG). Sections across control (B,F,J) and mutant (C,G,K) tongues at the level of the circumvallate papilla showing RALDH1-3 protein expression in von Ebner’s glands (vEG). vEGs in the mutants are relatively overgrown and exhibit mucous metaplasia (mucous cells are filled with a light blue material). Sections across the oral tongue of mutants (D,H,L) showing RALDH1-3 protein expression in the heterotopic lingual glands (HG). Scale bars: 100 μm (A-L). (TIF) [file pgen.1006914.s007.tif]

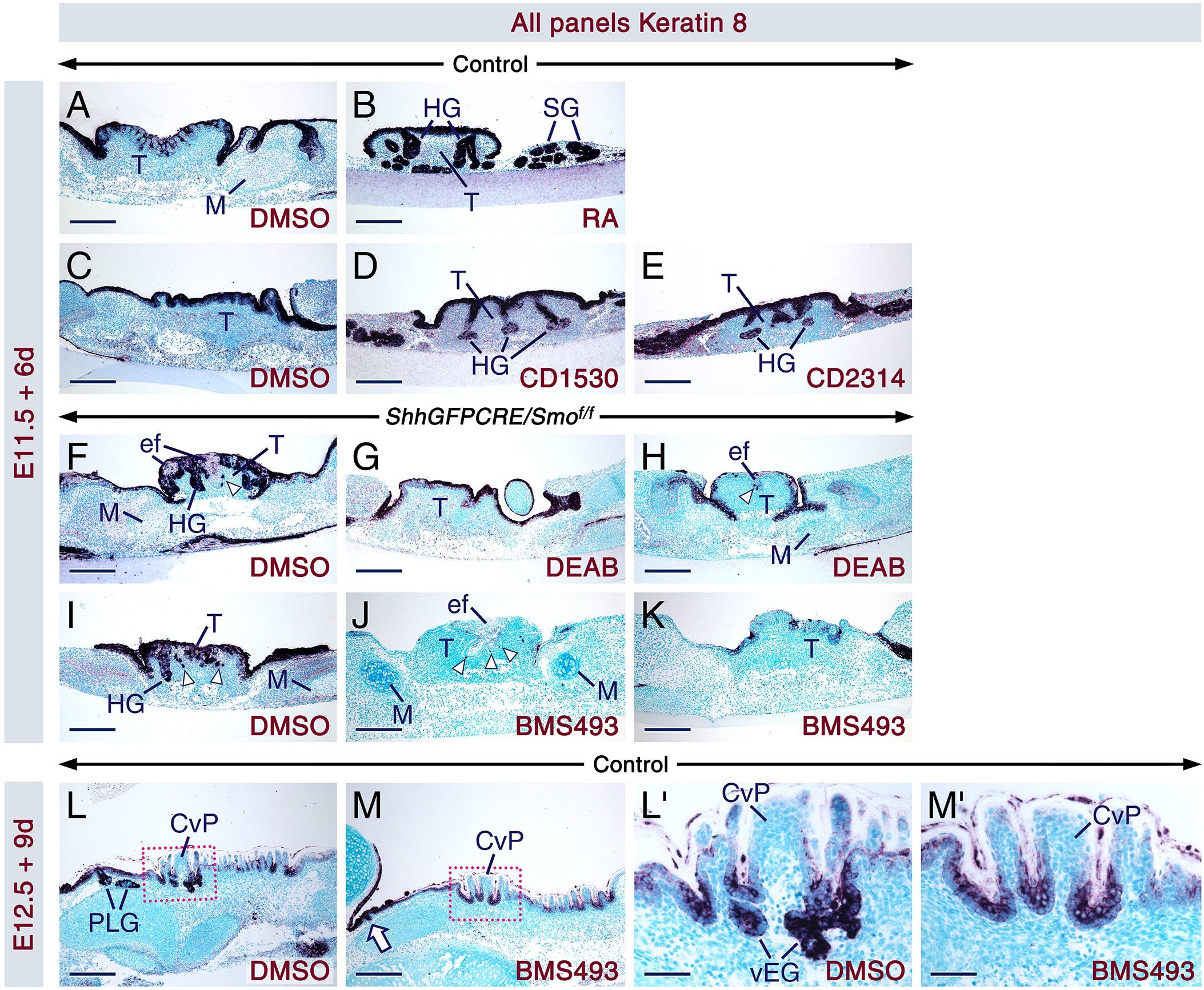

Supplement: S7 Fig — (A-K) Anti-Keratin 8 (K8)–stained (dark purple) frontal sections across the oral tongue (T) in E11.5 mandibular arch explants cultured in vitro for 6 days. (A-E) Explants from control embryos (without the CRE and/or the Smo floxed alleles) treated with vehicle (DMSO; A) and 3 μM all trans-retinoic acid (RA; B). Explants from control embryos treated with DMSO (C), 1 μM CD1530 (D) and 1 μM CD2314 (E). The retinoids induced glandular metaplasia, i.e. formation of heterotopic glands (HG) in the oral tongue, and promoted salivary gland (SG) formation, but they inhibited development of Meckel’s cartilage (M) and other mandibular structures. (F-K) Explants from E11.5 ShhGFPCRE/Smof/f mutants treated with DMSO (F,I), DEAB (G,H) and 12.5 μM BMS493 (J,K). DEAB was used at 20 μM and 10 μM during the first and last 3 days of culture, respectively. (H) and (K) are sections across the anterior-most part of the oral tongue of the specimens shown in (G) and (J), respectively. The DMSO-treated mutant tongues recapitulated the in vivo defects, including formation of K8-positive (+) heterotopic glands (HG), K8+ ectopic Merkel cells (arrowheads in F and I), and K8-negative (–) squamous epithelial foci (ef). DEAB and BMS493 inhibited heterotopic gland formation (G,H,J,K), but failed to inhibit Merkel cell metaplasia (arrowheads in H and J point at the ectopic Merkel cells) and formation of K8(–) epithelial foci. (L-M’) Anti-K8-stained oblique sections across the circumvallate papilla (CvP) and posterior lingual glands (pLG) of E12.5 mandible/tongue explants from control embryos after 9 days of in vitro culture in the presence of vehicle (DMSO; L) and 10 μM BMS493 (M). (L’) and (M’) are enlarged images of the boxed areas in (L) and (M), respectively. BMS493 inhibited development of posterior lingual glands (arrow in M) and von Ebner’s glands (vEG). Scale bars: 200 μm (A-K, L,M) and 50 μm (L’,M’). (TIF) [file pgen.1006914.s008.tif]

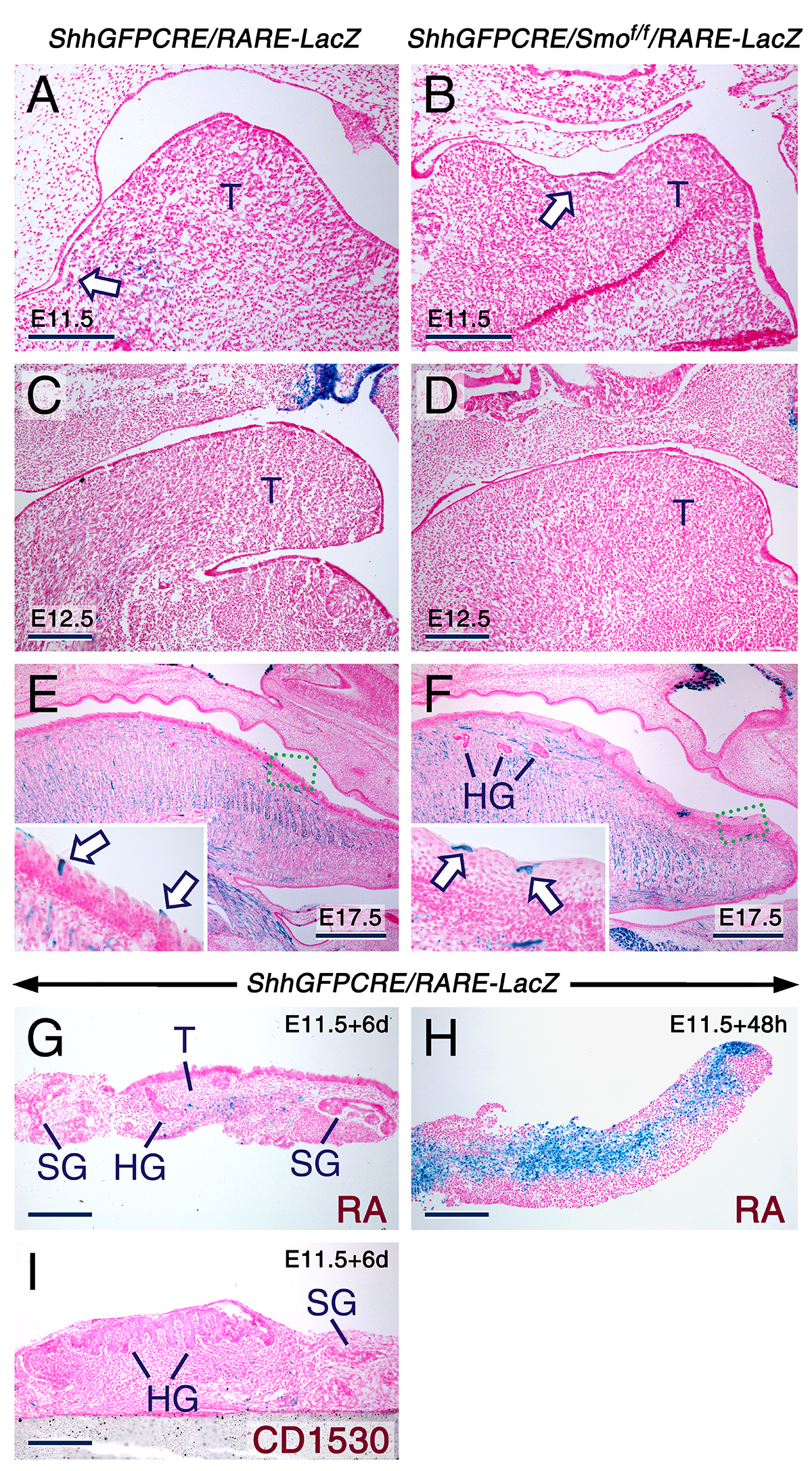

Supplement: S8 Fig — (A-I) β-galactosidase (β-gal) histochemistry for visualization of RARE-hsp68-LacZ transgene activity (blue) in cryostat sections. (A-F) Parasagittal tongue (T) sections from control (ShhGFPCRE/RARE-LacZ) and mutant (ShhGFPCRE/Smof/f/RARE-LacZ) embryos at E11.5 (A,B), E12.5 (C,D) and E17.5 (E,F). The insets in (E) and (F) are enlarged images of the boxed areas in (E) and (F), respectively, and show β-gal-positive (+) desquamating suprabasal epithelial cells. Weak β-gal activity in subsets of epithelial and mesenchymal cells at the junction between branchial arches 1& 2 (arrows in A and B). The quasi-totality of the lingual epithelium in the controls and mutants is β-gal-negative (A-F). The heterotopic glands (HG) in the mutant are β-gal-negative (F). (G-I) Sections across the oral tongue/mandible (G,I) and tail (H) from E11.5 ShhGFPCRE/RARE-LacZ embryos after in vitro culture for 6 (G,I) or 2 (H) days in the presence of 3 μM all-trans retinoic acid (RA; G,H) or 1 μM CD1530 (I). The tail explant was cultured in the same dish as the explant shown in (G). In contrast to the tail, neither the salivary glands (SG) nor the retinoid-induced heterotopic glands (HG) in the oral tongue are β-gal+. Scale bars: 500 μm (E,F) and 200 μm (A-D,G-I). (TIF) [file pgen.1006914.s009.tif]

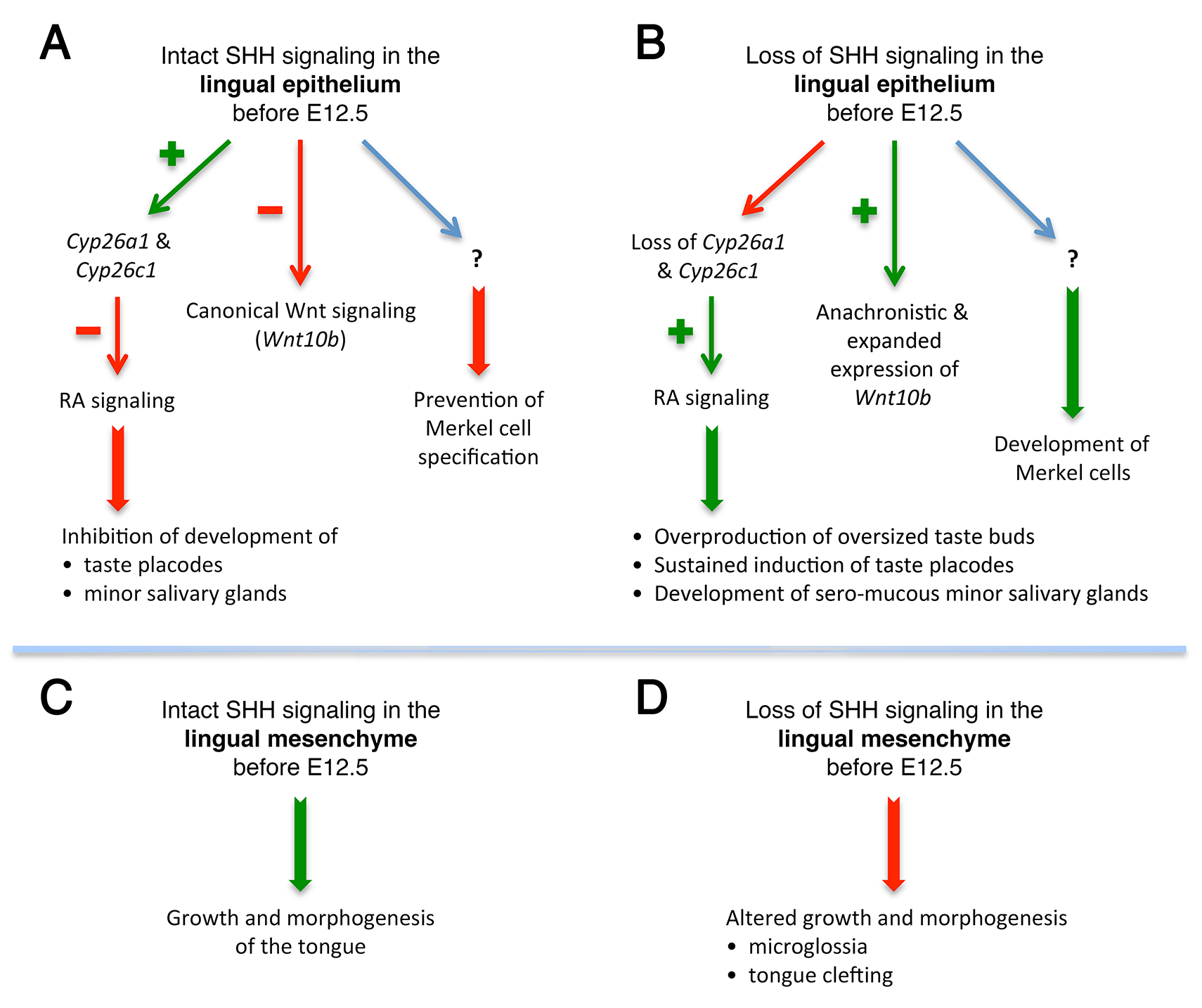

Supplement: S9 Fig — (A) Before E12.5, SHH signaling controls antero-posterior patterning and cell fate specification in the LE of the oral tongue, whereby SHH inhibits the development of minor salivary glands and taste placodes (thick red arrow). SHH fulfills this function by abating retinoic acid (RA) signaling (thin red arrow) in the interplacodal epithelium through maintenance and/or reinforcement of expression of transcripts encoding the RA catabolic enzymes CYP26A1 and CYP26C1 (thin green arrow). SHH input in the LE is also required to inhibit Merkel cell specification (thick red arrow) through a yet to be determined mechanism (blue arrow). SHH is known to antagonize canonical Wnt signaling (thin red arrow), a promoter of taste placode induction. This is achieved, at least in part, through inhibition of Wnt10b expression. (B) Loss of SHH signaling in the LE before E12.5 causes loss of expression of Cyp26a1 and Cyp26c1 expression (thin red arrow). Hence, unabated RA signaling (thin green arrow), triggered by the highly diffusible small molecule RA (not depicted), causes overproduction of oversized taste buds, sustained induction of taste placodes, and development of sero-mucous minor salivary glands (thick green arrow). Furthermore, absence of epithelial SHH signaling prompts the LE to aberrantly generate Merkel cells (thick green arrow), the underlying mechanism of which is unknown (blue arrow). Loss of epithelial SHH inputs also leads to anachronistic and expanded expression of Wnt10b (thin green arrow). (C) Before E12.5, SHH signaling in the lingual mesenchyme (LM) is crucial (green arrow) for growth and morphogenesis of the tongue but is not required for cell fate specification in the LE. (D) Loss of SHH signaling in the LM before E12.5 causes altered growth (microglossia) and morphogenesis (clefting) of the tongue (thick red arrow). (TIF) [file pgen.1006914.s010.tif]
