# Supplementary material for: Cell fate specification in the lingual epithelium is controlled by antagonistic activities of Sonic hedgehog and retinoic acid
Source: PLoS Genet. 2017 Jul 17;13(7):e1006914. doi: 10.1371/journal.pgen.1006914 (PMC5536368; doi:10.1371/journal.pgen.1006914)
Supplement: S1 Table — (PDF) [file pgen.1006914.s011.pdf]

S1 Table

Quantification of taste buds and ectopic Merkel cells in controls and *ShhGFPCRE/Smo*$^{f/f}$ tongues after immunofluorescence

Marker/genotype	Controls	*ShhGFPCRE/Smo*$^{f/f}$ mutants
	**Taste buds**	**Taste buds**
Keratin 8 (K8)/Sonic Hedgehog (SHH)	6 out of 6 (6/6) K8+ taste buds (TBs) were SHH+ (n=3)	41/41 K8+ TBs were SHH+ (n=5)
K8/Homer1	22/22 K8+ TBs were Homer1+ (n=3)	35/35 K8+ TBs were Homer1+ (n=3)
K8/Rab3c	11/11 K8+ TBs were Rab3c-negative (–) (n=3)	48/48 K8+ TBs were Rab3c (–) (n=5)
K8/P2X2	31/31 K8+ TBs were innervated by P2X2+ axons (n=4)	45/45 K8+ TBs were innervated by P2X2+ axons (n=4)
	**Ectopic Merkel cells**	**Ectopic Merkel cells**
K8/SHH	No K8+ ectopic Merkel cells (MCs) and no SHH+ single cells present in the epithelial basal layer (n=3)	113/113 K8+ ectopic MCs were SHH(–) (n=5)
K8/Homer1	No K8+ and no Homer1+ single cells present in the epithelial basal layer (n=3)	75/75 K8+ ectopic MCs were Homer1(–) (n=3)
K8/Rab3c	No K8+ and no Rab3c+ single cells present in the epithelial basal layer (n=3)	107/113 K8+ ectopic MCs were Rab3c+ (6/113 showed weak Rab3c staining) (n=5)
K8/P2X2	No K8+ single cells present in the epithelial basal layer (n=4)	95/95 K8+ ectopic MCs were not associated with any P2X2+ axons (n=4)
K8/NF-200	Controls not assessed	102/107 K8+ ectopic MCs were innervated by NF-200+ axons (n=4)
