# Supplementary material for: Cell fate specification in the lingual epithelium is controlled by antagonistic activities of Sonic hedgehog and retinoic acid
Source: PLoS Genet. 2017 Jul 17;13(7):e1006914. doi: 10.1371/journal.pgen.1006914 (PMC5536368; doi:10.1371/journal.pgen.1006914)
Supplement: S2 Table — (PDF) [file pgen.1006914.s012.pdf]

S2 Table

Quantification of taste buds innervated by gustatory neurites in controls and *ShhCreER*^{T2}*/Shh*^{f} mutants

**Marker/genotype**	**Controls at E18 (n=3) Tamoxifen-induced at E10.5 ***	***ShhCreER*^{T2}*/Shh*^{f} mutants at E18 (n=3) Tamoxifen-induced at E10.5 ****
Keratin 8/P2X2	22 out of 22 (22/22) K8+ taste buds (TBs) innervated by P2X2 axons	24/24 K8+ TBs innervated by P2X2 axons
**Marker/genotype**	**Controls at E18 (n=4) Tamoxifen-induced at E11.5 ***	***ShhCreER*^{T2}*/Shh*^{f} mutants at E18 (n=4) Tamoxifen-induced at E11.5 ***
Keratin 8/P2X2	45/45 K8+ TBs innervated by P2X2 axons	102/102 K8+ TBs innervated by P2X2 axons

* No basally located K8+ ectopic Merkel cells were observed

** 4 basally located K8+ ectopic Merkel cells were observed; none was invested by P2X2 neurites
