# Supplementary material for: Cell fate specification in the lingual epithelium is controlled by antagonistic activities of Sonic hedgehog and retinoic acid
Source: PLoS Genet. 2017 Jul 17;13(7):e1006914. doi: 10.1371/journal.pgen.1006914 (PMC5536368; doi:10.1371/journal.pgen.1006914)
Supplement: S3 Table — (PDF) [file pgen.1006914.s013.pdf]

S3 Table

Retinoids induce lingual glandular metaplasia and retinoic acid signaling inhibitors abrogate heterotopic and orthotopic lingual gland formation

**E11.5 tongue explants after 6 days of *in vitro* culture**		
**Explants**	**Treatment/number of explants**	**Outcome**
Controls*	DMSO control for *all-trans* retinoic acid (atRA) (n=5)	*** Expected outcome of cultured explants in 5/5
Controls*	atRA at 3 µM (n=5)	Glandular metaplasia (GM) in 5/5 explants
Controls*	DMSO** control for CD2314 (n=2) and CD1530 (n=2)	*** Expected outcome of cultured explants in 4/4
Controls*	1 µM CD2314 (n=2)	GM in 2/2 explants
Controls*	1 µM CD1530 (n=2)	GM in 2/2 explants
ShhGFPCRE/Smo$^{f/f}$ mutants	DMSO control for BMS493 at 10 µM (n=1) and 12.5 µM (n=1)	GM and Merkel cell metaplasia (MCM) in 2/2 explants
ShhGFPCRE/Smo$^{f/f}$ mutants	10 µM (n=5) and 12.5 µM (n=6) BMS493	Inhibition of GM but not MCM in 11/11 explants
ShhGFPCRE/Smo$^{f/f}$ mutants	DMSO control for DEAB (n=2)	GM and MCM in 2/2 explants
ShhGFPCRE/Smo$^{f/f}$ mutants	20 µM and 10 µM DEAB the first and last 3 days, respectively (n=6)	Inhibition of GM but not MCM in 6/6 explants
Controls*	DMSO control for BMS493 at 10 µM (n=4) and 12.5 µM (n=4)	*** Expected outcome of cultured explants in 8/8
Controls*	10 µM (n=8) and 12.5 µM (n=8) BMS493	*** Expected outcome of cultured explants in 16/16
Controls*	DMSO control for DEAB (n=1)	*** Expected outcome of cultured explants in 1/1
Controls*	20 µM and 10 µM DEAB the first and last 3 days, respectively (n=14)	*** Expected outcome of cultured explants in 14/14
**E12.5 tongue explants after 9 days of *in vitro* culture**		
**Explants**	**Treatment/number of explants**	**Outcome**
Controls*	DMSO control for BMS493 (n=4)	Development of von Ebner's glands in 3/4 explants (one specimen was inconclusive due to loss of sections at the level of the circumvallate papilla) and posterior lingual glands in 4/4 explants
Controls*	10 µM BMS493 (n=4)	Inhibition of von Ebner's gland and posterior lingual gland formation in 4/4 explants

atRA, all-trans retinoic acid; GM, glandular metaplasia; MCM, Merkel cell metaplasia. * The Controls include explants from embryos lacking the *ShhGFPCRE* and/or the floxed *Smo* alleles. ** The same final concentration of DMSO was used as control for CD2314 and CD1530 since the stock solutions of both compounds were at 25 mM. *** Taste buds, innervation, vascularization and muscles do not develop in cultured explants.
