# Supplementary material for: Cell fate specification in the lingual epithelium is controlled by antagonistic activities of Sonic hedgehog and retinoic acid
Source: PLoS Genet. 2017 Jul 17;13(7):e1006914. doi: 10.1371/journal.pgen.1006914 (PMC5536368; doi:10.1371/journal.pgen.1006914)
Supplement: S4 Table — (PDF) [file pgen.1006914.s014.pdf]

S4 Table

Retinoids promote and inhibitors of retinoic acid signaling inhibit fungiform placode formation after a 2-day treatment of E12 tongue explants

**Explants**	**Treatment/number of explants**	**Outcome**
Controls*	DMSO control for *all-trans* retinoic acid (atRA) (n=2)	Development of *Shh+* spots in 2/2 explants
Controls*	atRA at 3 µM (n=2)	Development of enlarged *Shh+* spots in 2/2 explants
Controls*	DMSO** control for CD2314 and CD1530 at 0.5 µM (n=7)	Development of *Shh+* spots in 7/7 explants
Controls*	CD2314 (n=2) or CD1530 (n=3) at 0.5 µM	Development of enlarged *Shh+* spots in 5/5 explants
Controls*	DMSO** control for CD2314 and CD1530 at 1 µM (n=6)	Development of *Shh+* spots in 6/6 explants
Controls*	CD2314 (n=10) and CD1530 (n=7) at 1 µM	Development of enlarged *Shh+* spots in 17/17 explants and abnormal *Shh* expression along the tongue's midline in 7/7 CD1530-treated explants
Controls*	DMSO** control for CD2314 and CD1530 at 1.5 µM (n=4)	Development of *Shh+* spots in 4/4 explants
Controls*	CD2314 (n=2) and CD1530 (n=5) at 1.5 µM	Development of enlarged *Shh+* spots, including in two domains flanking the prospective intermolar eminence, and abnormal expression of *Shh* along the tongue's midline in 7/7 explants
Controls*	DMSO control for BMS493 at 12.5 µM (n=5)	Development of *Shh+* spots in 5/5 explants
Controls*	BMS493 at 12.5 µM (n=12)	Development of large areas devoid of *Shh+* spots in 10/12 explants
ShhGFPCRE/Smo$^{f/f}$ mutants	DMSO*** control for BMS493 at 12.5 µM or DEAB at 25 µM (n=4)	Expansion of *Shh* expression domains in 4/4 explants, phenocopying the *in vivo* anomaly
ShhGFPCRE/Smo$^{f/f}$ mutants	BMS493 at 12.5 µM (n=1)	Development of large areas devoid of *Shh+ spots*
ShhGFPCRE/Smo$^{f/f}$ mutants	DEAB at 25 µM (n=4)	Development of large areas devoid of *Shh+* spots in 4/4 explants

atRA, all-trans retinoic acid; *Shh,* Sonic hedgehog. * The control explants used were from embryos lacking the *ShhGFPCRE* and/or the floxed *Smo* alleles. ** The same final concentration of DMSO was used as control for CD2314 and CD1530 since the stock solutions of both compounds were at 25 mM. *** The same final concentration of DMSO was used as control for BMS493 and DEAB since the stock solutions were at 50 mM and 100 mM, respectively.
